# Adenosine A1 receptor: A neuroprotective target in light induced retinal degeneration

**DOI:** 10.1371/journal.pone.0198838

**Published:** 2018-06-18

**Authors:** Manuel Soliño, Ester María López, Manuel Rey-Funes, César Fabián Loidl, Ignacio M. Larrayoz, Alfredo Martínez, Elena Girardi, Juan José López-Costa

**Affiliations:** 1 Universidad de Buenos Aires, Facultad de Medicina, Dpto. de Biología Celular, Histología, Embriología y Genética, Ciudad Autónoma de Buenos Aires, Argentina; 2 CONICET-Universidad de Buenos Aires. Instituto de Biología Celular y Neurociencia “Prof. E. De Robertis¨ (IBCN), Ciudad Autónoma de Buenos Aires, Argentina; 3 Biomarkers and Molecular Signaling Group, Center for Biomedical Research of La Rioja (CIBIR), Logroño, Spain; 4 Angiogenesis Study Group, Center for Biomedical Research of La Rioja (CIBIR), Logroño, Spain; University of Florida, UNITED STATES

## Abstract

Light induced retinal degeneration (LIRD) is a useful model that resembles human retinal degenerative diseases. The modulation of adenosine A1 receptor is neuroprotective in different models of retinal injury. The aim of this work was to evaluate the potential neuroprotective effect of the modulation of A1 receptor in LIRD. The eyes of rats intravitreally injected with N6-cyclopentyladenosine (CPA), an A1 agonist, which were later subjected to continuous illumination (CI) for 24 h, showed retinas with a lower number of apoptotic nuclei and a decrease of Glial Fibrillary Acidic Protein (GFAP) immunoreactive area than controls. Lower levels of activated Caspase 3 and GFAP were demonstrated by Western Blot (WB) in treated animals. Also a decrease of iNOS, TNFα and GFAP mRNA was demonstrated by RT-PCR. A decrease of Iba 1^+^/MHC-II^+^ reactive microglial cells was shown by immunohistochemistry. Electroretinograms (ERG) showed higher amplitudes of a-wave, b-wave and oscillatory potentials after CI compared to controls. Conversely, the eyes of rats intravitreally injected with dipropylcyclopentylxanthine (DPCPX), an A1 antagonist, and subjected to CI for 24 h, showed retinas with a higher number of apoptotic nuclei and an increase of GFAP immunoreactive area compared to controls. Also, higher levels of activated Caspase 3 and GFAP were demonstrated by Western Blot. The mRNA levels of iNOS, nNOS and inflammatory cytokines (IL-1β and TNFα) were not modified by DPCPX treatment. An increase of Iba 1^+^/MHC-II^+^ reactive microglial cells was shown by immunohistochemistry. ERG showed that the amplitudes of a-wave, b-wave, and oscillatory potentials after CI were similar to control values. A single pharmacological intervention prior illumination stress was able to swing retinal fate in opposite directions: CPA was neuroprotective, while DPCPX worsened retinal damage. In summary, A1 receptor agonism is a plausible neuroprotective strategy in LIRD.

## Introduction

Human retinal degenerative diseases are important disabling conditions. Among them, age-related macular degeneration (AMD) is the first cause of acquired blindness in developed countries [1]. In the US, the prevalence of AMD is similar to that of all invasive cancers combined and more than double the prevalence of Alzheimer´s disease [2]. The treatment of advanced neovascular AMD (“wet¨ variant) consists mainly on the use of monoclonal antibodies against vascular endothelial growth factor (VEGF) but the ¨dry¨ variant of AMD has no reliable treatment yet. Current treatments for dry AMD slow down or prevent additional vision loss to some extent but they do not restore lost vision. The majority of patients require indefinite treatment or demonstrate disease progression despite therapies [2]. A meta-analysis shows that 20–25% of unilateral AMD cases, and up to 50% of unilateral late AMD cases progress to bilateral in 5 years [3]. These evidences show the importance of exploring other pharmacological tools to deal with retinal degenerative diseases. Recent articles have also shown the neuroprotective effect of peptides such as pituitary adenylate cyclase-activating peptide (PACAP) and the octapeptide NAP, derived from activity-dependent neuropeptide protein (ADNP), in rat diabetic retinopathy which counteract the up-regulation of VEGF [4, 5].

Animal models of retinal degenerative diseases must be employed to test potential pharmacological treatments. Light induced retinal degeneration (LIRD) has been widely used to study degenerative diseases of the retina [6–12]. The main hallmarks of the LIRD model are similar to some of those detected in human AMD, juvenile macular degeneration or retinitis pigmentosa. The degenerative process starts in the outer retina as continuous illumination (CI) produces photoreceptor (PH) degeneration, apoptosis in the outer nuclear layer (ONL), increased phagocytosis by the retinal pigment epithelium (RPE) and synaptic degeneration in the outer plexiform layer (OPL) [7, 13–17]. Conversely, in other degenerative diseases such as diabetic retinopathy, retinopathy of prematurity, glaucoma, and ischemia, degeneration starts in the inner retina and affects primarily to inner nuclear layer, ganglion cell layer and optic nerves [18–20].

In our hands, treating albino rats (Sprague Dawley) with white light (12 klux) produces a peak of NO after one day of continuous illumination [10], an increase of glucocorticoids, a great number of apoptotic nuclei in the outer nuclear layer after 2 days of continuous illumination [7], and the complete loss of photoreceptors after 7 days of continuous illumination [17].

Adenosine is a non-classical transmitter found in the extracellular space as a consequence of ATP breakdown by ectonucleosidases or through translocation by membrane nucleoside transporters. Adenosine binds to G protein coupled receptors belonging to the P1 family of receptors known as A1, A2A, A2B and A3 receptors [21, 22]. Different autoradiographic and *in situ* hybridization studies have shown the localization of adenosine receptors in the retina of rabbits, mice, rats, monkeys, and humans [23–26].

In recent years, the modulation of adenosine receptors has emerged as a potential neuroprotective strategy to treat a wide range of insults and degenerative diseases of the CNS [27]. A1 receptor agonists have been reported to be neuroprotective in animal models of epilepsy, inflammatory, hypoxic, and degenerative diseases of the CNS [28–30]. In humans with Alzheimer´s disease, A1R expression rises and is associated with number of amyloid plaques and Tau phosphorylation. It was suggested that adenosine could slow down the progression of Alzheimer´s disease [31].

Adenosine release is an important component of the ischemic/hypoxic insult to the retina [32, 33], where it probably produces hyperhemia that protects neurons from glutamate toxicity [34]. The neuroprotective role of adenosine after the ischemic injury of the retina is mediated via A1R and/or A2R [35]. Furthermore, recent works have demonstrated the neuroprotective role of A2A receptor antagonists against damage induced by retinal ischemia both in animal models of ischemia-reperfusion and in primary microglial cultures submitted to elevated hydrostatic pressure [36, 37]. Although there is an extensive knowledge about the neuroprotective role of adenosine in different models of retinal degenerations, including ischemic and diabetic retinopathy [38], little is known about the role of adenosine in degenerative diseases of the outer retina.

In order to improve our knowledge on the processes underlying light induced retinal degenerations, and as a first step to assess new potential therapeutic targets, the role of A1R in the degenerative process was studied by modulating its activity with an A1R agonist (cyclopentyladenosine -CPA-) or an A1R antagonist (dipropylcyclopentylxanthine -DPCPX-) in the LIRD model. The effects of these drugs were studied by Terminal deoxynucleotidyl transferase dUTP nick end labeling (TUNEL) and activated Caspase-3 Western Blotting (WB), and their effects on glial reactivity were determined by Glial Fibrillary Acidic Protein (GFAP) immunohistochemistry, Western Blot and qRT-PCR. Changes in microglia were studied by Iba1 (ionized calcium binding adaptor molecule 1) and major histocompatibility complex class II (MHC-II) immunohistochemistry. The effects of these drugs on retinal physiology were determined by electroretinography (ERG). In order to know the mechanisms involved in A1R modulatory effect of light induced retinal degeneration, the expression of inflammatory cytokines, iNOS, and nNOS genes was explored by qRT-PCR.

## Materials and methods

### Animals

56 Male Sprague Dawley albino rats (body weight 200g, age 60 days) were used. Rats were obtained from the animal house of the Facultad de Veterinaria, Universidad de Buenos Aires. Before the pharmacological treatment by intravitreal injections, animals were kept at 12/12 h light/dark cycles (Lighting level: 80 lux during light period). Animal care was performed in accordance with the European Community Directive 2010/63/EU of 22 September 2010. The animal model of continuous illumination and the experimental procedure was approved by the Institutional Committee for the Use and Care of Laboratory Animals of the Facultad de Medicina, Universidad de Buenos Aires (“Comité Institucional para el Cuidado y Uso de Animales de Laboratorio”, CICUAL, Res. (CD) 2599/2013).

### Experimental design

**Fig 1:**
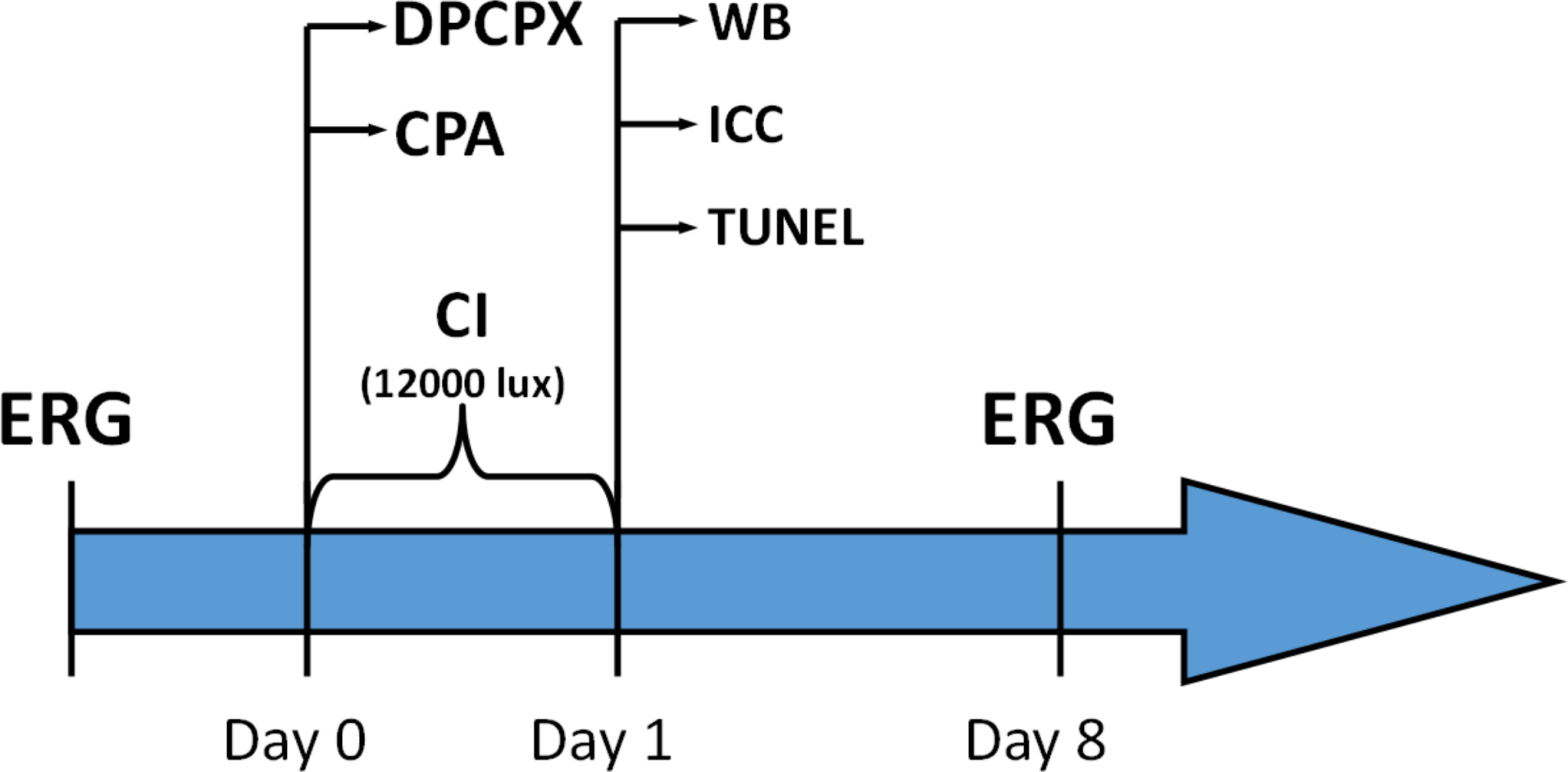
Timescale of the continuous illumination procedure. All animals were subjected to intravitreal injections of CPA or DPCPX on right eye and of vehicle on the left eye. After recovery, animals were continuously illuminated for one day (12000 lux). A group of animals was sacrificed right after the end of CI and they were processed for either IHC, TUNEL or WB assays. A second group of animals, which had been tested through a basal ERG, was left to recover for a week after CI and a follow up ERG was performed then.

Male Sprague Dawley albino rats were intravitreally injected with either cyclopentyladenosine (CPA), an A1R agonist; or with dipropylcyclopentylxanthine (DPCPX), an A1R antagonist. While the right eyes received the mentioned drugs, the left eyes received vehicle (CPA vehicle: 0.9% ClNa w/v in water; DPCPX vehicle: 0.3% DMSO v/v dil in 0.9% NaCl w/v in water) and were the controls. One hour after intravitreal injections, rats were continuously illuminated for 1 day (12000 lux). Then the retinas were processed for GFAP immunohistochemistry (IHC), TUNEL or Western Blotting (WB). Electroretinograms (ERG) were performed previous to intravitreal injections of drugs and also a week after continuous illumination (Fig 1).

### Intravitreal injections protocol

Animals were deeply anestethized with Ketamine (40mg/kg; Ketamina 50®, Holliday-Scott SA, Beccar, Argentina) and Xylazine (5 mg/kg; Kensol®, Laboratorios König SA., Buenos Aires, Argentina). A drop of 2% lidocaine (Lidocaine® Richmond división veterinaria SA, Grand Bourg, Buenos Aires, Argentina) was administered in each eye for local anaesthesia. Intravitreal injections (volume: 5 μl) were performed using a Hamilton syringe (Reno, NV, USA) and a 30-gauge needle. The right eyes received the studied drugs (either cyclopentyladenosine (CPA), an A1R agonist; or dipropylcyclopentylxanthine (DPCPX), an A1R antagonist) while the left eyes received vehicle and were the controls (CTL). The final vitreal concentrations achieved were 0.775 mM for CPA and 0.01 mM for DPCPX. Doses were selected based on previous scientific reports [39, 40] and taking into account that the volume of vitreous of the rat eye is 13.36 ± 0.64 μl [41]. The total amount per eye of CPA and DPCPX injected were 10.35 nanomoles and 0.13 nanomoles, respectively. To promote a correct healing, an ocular re-epithelization ointment (Oftalday®, Holliday-Scott SA, Beccar, Buenos Aires; Argentina) was applied after the injection. After recovery from the procedure, the animals were exposed to 1 day of CI.

### Continuous illumination procedure

One hour after intravitreal injections, rats were continuously illuminated for 1 day. Groups of 3 to 5 rats were simultaneously placed in an open white acrylic box of 60 cm x 60cm x 60cm with 12 halogen lamps (12V 50 W each) located on top. Lighting level (12,000 lux) was determined using a digital illuminance meter. Temperature was maintained at 21°C. This was repeated to obtain at least 8 animals for IHC, 4 animals for Western Blot procedures, 5 animals for ERG and 5 animals for qRT-PCR. IHC and Western Blot were performed immediately after CI. Animals used for ERG studies received a basal ERG previous to intravitreal injections of drugs and a second ERG (follow up) a week after CI (Fig 1). All animals were offered food and water *ad libitum*.

### Electroretinography

After overnight adaptation, rats (5 animals per drug treatment) were anesthetized under dim red illumination with Ketamine (40mg/kg; Ketamina 50®, Holliday-Scott SA, Beccar, Argentina) and Xylazine (5 mg/kg; Kensol®, Laboratorios König SA., Buenos Aires, Argentina). An ophthalmic solution of Phenylephrine hydrochloride 5% and tropicamide 0.5% (Fotorretin®, Laboratorios Poen, CABA, Argentina) was used to dilate the pupils. Rats were placed facing the stimulus at a distance of 25 cm in a highly reflective environment. A reference electrode was placed in the ear, a grounding electrode was attached to the tail, and a gold electrode was placed in contact with the central cornea. Recordings were made from both eyes simultaneously.

Scotopic electroretinograms (ERGs): 20 responses to flashes of unattenuated white light (1 ms, 1 Hz) from a photic stimulator (light-emitting diodes) set at maximum brightness were recorded with electroretinograph Akonic BIO-PC, Buenos Aires, Argentina. The registered response was amplified, filtered (1.5-Hz low-pass filter, 500Hz high-pass filter, notch activated) and data were averaged. The a-wave was measured as the difference in amplitude between the recording at onset and the through of the negative deflection while the b-wave amplitude was measured from the trough of the a-wave to the peak of the b-wave. Mean values from each eye were averaged, and the resultant mean value was used to compute the group means a- and b-wave amplitudes ± SD.

Oscillatory potentials (OPs): Briefly, the same photic stimulator was used with filters of high (300 Hz) and low (100 Hz) frequency. The amplitudes of the OPs were estimated by using the peak-to-trough method [42]. The sum of four OPs was used for statistical analysis.

### SDS-PAGE and Western-blotting

Retinas of CPA (n = 4) and DPCPX (n = 4) treated animals were dissected out. Five control retinas were used on each case (n = 5). Tissues were homogenized (1:3, w/v) in lysis buffer (100 mM NaCl, 10 mM TrisHCL, 0.5% Triton X-100) plus 50ul of Protease inhibitor cocktail (Merck KGaA, Darmstadt, Germany). All procedures were carried out at 4°C. Protein concentration was determined by the Bradford method, with bovine serum albumin as standard, using a Beckman Spectrophotometer DU-65. Then, 50–100 μl of each sample were mixed 4:1 with 5X sample buffer (10% SDS, 0315 M Tris-HCl, 25% Beta-Mercaptoethanol, 50% Glycerol, 0.2 ml bromophenol blue 0.1%, pH 6.8) and heated for 10 minutes at 100°C. Samples were run (50 μg of protein per lane) on SDS–polyacrylamide gels (10% or 15% running gels with 5% stacking gel), with 0.24 mM TRIS base, 4.38 mM SDS, 0.19 M glycine, pH 8.3, as the electrolyte buffer. Kaleidoscope Prestained Standards (Bio-Rad Laboratories, California, USA) were used as molecular weight markers. For Western Blot analysis, proteins were transferred at 100 mVolt for 1 h onto 0.2-μm polyvinylidenedifluoride membranes (GE healthcare life sciences, Illinois, USA) in a transfer buffer (15% m/v Glycine, 3% m/v TRIS, 20% v/v ethanol).

Membranes were incubated overnight at 4°C with either a rabbit polyclonal antibody to GFAP (DAKO Inc., CA, USA; dilution 1:500) or a rabbit polyclonal antibody to activated Caspase 3 (Sigma Chemical Co., MO., USA; dilution 1:100). To test for protein loading accuracy, a monoclonal anti-β-actin antibody (Sigma Chemical Co., MO., USA, dil: 1: 1000) was used in the same membranes. To visualize immunoreactivity, membranes were incubated with Amersham ECL Rabbit IgG, HRP-linked F(ab)2 fragment (from donkey), and were developed using a chemoluminiscence kit (SuperSignal West Pico Chemiluminescent Substrate, Thermo Scientific, Massachusetts, US). Membranes were exposed to X-ray blue films (Agfa Heathcare, Buenos Aires, Argentina), which were developed and then scanned with a HP Photosmart scanner. Optical density was quantified by Image Studio Light software of Li-Cor. Relative density is compared to control levels. Differences in actin load were taken in consideration in each case and data were mathematically corrected in order to obtain the published results. Data were statistically analysed using Graphpad Software.

### Tissue processing for immunohistochemistry and TUNEL assay

Animals were deeply anaesthetized by intraperitoneal injection of Ketamine (40mg/kg; Ketamina 50®, Holliday-Scott SA, Beccar, Argentina) and Xylazine (5 mg/kg; Kensol®, Laboratorios König SA., Buenos Aires, Argentina) and their eyes were removed; the cornea and lenses were cut off, and the remaining tissues with a cup shape were fixed by immersion in a solution containing 4% paraformaldehyde in 0.1M phosphate buffer for 24 h. Eyes were embedded in gelatine, cryoprotected by immersion in a solution containing 30% sucrose in 0.1M phosphate buffer and then frozen. The frozen eyes were cut along a vertical meridional plane using a Lauda Leitz cryostat, and sections (thickness: 20 μm) were mounted on gelatine coated glass slides and processed by Immunoperoxidase, immunofluorescence or TUNEL techniques.

### Immunoperoxidase technique

In order to inhibit endogenous peroxidase activity, sections were incubated in methanol containing 3% hydrogen peroxide for 30 min. After washing in phosphate buffered saline (PBS), pH 7.4, sections were incubated in 10% normal goat serum for 1h. Then, sections were incubated overnight with a previously characterized GFAP polyclonal primary antibody (Dako, USA, dilution 1:500). The following day, sections were incubated in biotinylated goat anti rabbit antibody (Sigma Chemical Co.,MO., USA; dilution 1:500). Following this, sections were incubated in ExtrAvidin-Peroxidase® complex (Sigma Chemical Co., MO., USA; dilution 1:500). All antisera were diluted in phosphate-buffered saline (PBS) containing 0.2% Triton X-100 and, in all but in the peroxidase complex, 1% normal goat serum. Incubations in primary antibody were performed overnight at 4°C while incubations in biotinylated antibody, ExtrAvidin-Peroxidase® complex were performed at room temperature (RT) for 1h. Controls were performed by omitting primary antibodies. Development was performed using the DAB/nickel intensification procedure [43].

### Immunofluorescence technique

Sections were incubated overnight with an Iba1 rabbit polyclonal antibody (Thermo Fisher Scientific Inc., USA, dilution 1:125). The following day, sections were incubated in biotinylated goat anti rabbit antibody (Sigma Chemical Co.,MO., USA; dilution 1:125), and later in Streptavidin-Alexa Fluor® 635 conjugate (Thermo Fisher Scientific Inc., USA, dilution 1:50). Incubations in biotynilated goat anti rabbit antibody and Streptavidin-Alexa Fluor® 635 conjugate were performed at RT for 1 h. Finally, sections were counterstained with Hoechst 33258 (Sigma Chemical Co., MO., USA) and were observed using an Olympus IX-81 inverted microscope.

### Double labelling technique

Some sections were incubated overnight with a mixture containing a polyclonal rabbit antibody to A1R (Santa Cruz Biotech. Inc., USA, dilution 1:50) and a mouse monoclonal antibody to Iba 1 (Santa Cruz Biotech. Inc., USA, dilution 1:50). Other sections were incubated overnight with a mixture containing a mouse monoclonal to major histocompatibility complex class II (MHC-II) (Santa Cruz Biotech. Inc., USA, dilution 1:50) and a rabbit polyclonal antibody to Iba 1 (Invitrogen USA, dilution 1:50).

In both cases sections were later incubated in a mixture of goat anti rabbit antibody conjugated to Alexa Fluor® 488 (Abcam, dilution 1:50) and goat anti-mouse antibody conjugated to Alexa Fluor® 555 (Abcam, dilution 1:50) at RT for 1 h. Finally, sections were counterstained with Hoechst 33258 (Sigma Chemical Co., MO., USA) and were observed using an Olympus IX-83 inverted microscope. Simultaneously, negative controls were performed by omitting primary antibodies and their photographs were added to S1 Fig.

### Terminal deoxynucleotidyl transferase dUTP nick end labeling (TUNEL) assay

Cryostat sections were processed using the ApopTag® Peroxidase In Situ kit (Millipore, USA). Briefly, sections were washed in PBS and post-fixed in ethanol:acetic acid (2:1) at -20°C. After washing in PBS the endogenous peroxidase was quenched with 3% hydrogen peroxide solution at RT. After rinsing with distilled water and equilibration buffer, sections were incubated with terminal deoxynucleotidyl transferase for 1 hour at 37°C. The reaction was stopped by a supplied buffer and the sections were incubated with anti-digoxigenin conjugate for 30 minutes at RT. Finally sections were developed using DAB/nickel intensification procedure and were counterstained with eosine.

### Image analysis of TUNEL, GFAP immunoperoxidase sections and single or double labeled microglial cells

Six retinal sections of both eyes from each experimental group were analyzed (CPA, n = 8; DPCPX n = 8). Care was taken on selecting anatomically matched areas of retina among animals before assays. Slides were analysed using a Zeiss Axiophot microscope attached to a video camera (Olympus Q5). Images were taken using Q capture software. To avoid external variations, all images were taken the same day and under the same light conditions.

The following parameters were measured, blind to treatment, on 8 bits images, using the Fiji software (NIH, Research Services Branch, NIMH, Bethesda, MD):

*GFAP positive area*: Images of drug treated and control retinas were randomly selected. Immunoreactive area of the whole sections was thresholded. The region of interest (ROI) was the retinal surface between the two limiting membranes where Müller cells extend their processes. The GFAP positive area was calculated as the percentage of the ROI immunostained by GFAP.

*TUNEL positive nuclei/1000*μ*m*^*2*^: Images of drug treated and control retinas were randomly selected and thresholded. As region of interest (ROI), frames of 1000 μm^2^ were randomly determined on the outer nuclear layer of treated and control retinas. The analyse particles function of Fiji was used [44] and the TUNEL positive nuclei/1000μm^2^ ratio was then obtained in each ROI.

*Iba 1 positive microglial cells/10000*μ*m*^*2*^: Images of drug treated and control retinas were randomly selected and thresholded. As region of interest (ROI), frames of 10000 μm^2^ were randomly determined on treated and control retinas. The Iba 1 positive microglial cells/10000μm^2^ ratios were obtained in each ROI.

*Iba 1*^*+*^ */MHC-II*^*+*^ *microglial cells*. Images of drug treated and control retinas were quantified. The number of activated microglia (double labelled as Iba 1^+^ and MHC-II^+^ and) was expressed as the percentage of the total number of Iba 1 positive cells per retinal section.

### RNA isolation and quantitative reverse transcription polymerase chain reaction (qRT-PCR)

Unilluminated rats (basal control), rats submitted to 1 day of CI, CPA and DPCPX treated rats (n = 5 per group) which were submitted to one day of continuous illumination were deeply anaesthetized by intraperitoneal injection of Ketamine (40mg/kg; Ketamina 50®, Holliday-Scott SA, Beccar, Argentina) and Xylazine (5 mg/kg; Kensol®, Laboratorios König SA., Buenos Aires, Argentina) and their retinas were dissected out. In the cases of drug treated rats, right eyes received the studied drugs (either CPA or DPCPX), while the left eyes received vehicle and were the Controls (CTL). Additional controls were included: Non-illuminated control rats to evaluate basal gene level expression, and Non-treated rats (CTL) exposed to CI (CI 1d) in order to evaluate the effect of damage (n = 6, per group). Tissues were homogenized with TRIzol (Invitrogen, Madrid, Spain) and RNA was isolated with RNeasy Mini kit (Qiagen, Germantown, MD). Three μg of total RNA were treated with 0.5 μl DNAseI (Invitrogen) and reverse-transcribed into first-strand cDNA using random primers and the SuperScript III kit (Invitrogen). Reverse transcriptase was omitted in control reactions, where the absence of PCR-amplified DNA confirmed lack of contamination from genomic DNA. Resulting cDNA was mixed with SYBR Green PCR master mix (Invitrogen) for qRT-PCR using 0.3 μM forward and reverse oligonucleotide primers. Quantitative measures were performed using a 7300 Real Time PCR System (Applied Biosystems, Carlsbad, CA). Cycling conditions were an initial denaturation at 95ºC for 10 min, followed by 40 cycles of 95°C for 15 seconds and 60°C for 1 minute. At the end, a dissociation curve was implemented from 60 to 95ºC to validate amplicon specificity. Gene expression was calculated using absolute quantification by interpolation into a standard curve. All values were divided by the expression of the house keeping gene 18S.

### Statistical analysis

The data of GFAP immunohistochemistry and TUNEL studies of CPA-treated rats (n = 8) and DPCPX treated rats (n = 8) were obtained by image analysis as was described before. Normality distribution of the data was evaluated using D´Agostino, KS, Shapiro-Wilk and F tests. In every case, Gaussian distribution was confirmed. Then, data were analysed using unpaired parametric Student´s t-test included in the GraphPad software (GraphPad Software, San Diego, CA). Values are expressed as mean ± standard deviation. In the case of Iba 1 immunohistochemistry (IHC) (n = 4 per group), WB (n = 4 per group), ERG (n = 5 per group) and RT-PCR (n = 5 for CPA and DPCPX; n = 6 for CTL and CI 1d), data distribution was analysed in the same way and at least one of the used tests confirmed Gaussian distribution, validating the use of Student´s T test. Values are expressed as mean ± standard deviation. Differences were considered significant when p<0.05.

## Results

### CPA decreases apoptotic cell death, glial reactivity and Iba 1^+^/MHC- II^+^ microglial cells

**Fig 2:**
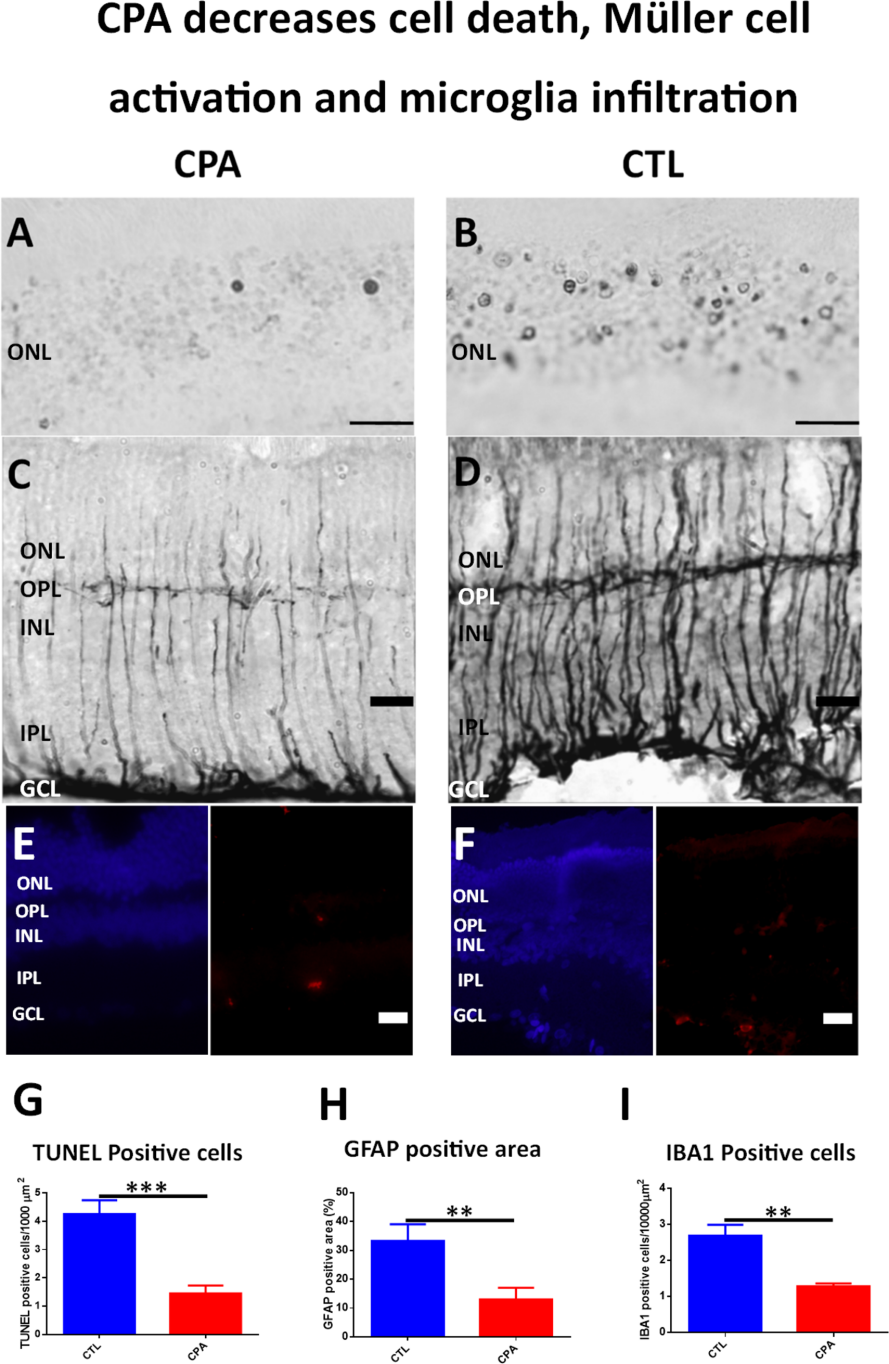
CPA treatment decreases cell death, Müller cell activation and microglial infiltration. A-B) Microphotograph of representative sections showing TUNEL staining of the outer nuclear layer (ONL) of CPA treated eye (A) and Control eye (B). Only two positive apoptotic nuclei may be observed in the field of CPA treated eye (A) while a huge number of apoptotic nuclei are observed in CTL eye (B). Scale bar: 20μm. C-D) Microphotograph of GFAP immunostained sections of CPA treated eye (C) and Control eye (D). Thin processes of Müller cells are observed in the retina of CPA treated eye (C) while thicker processes of Müller cells are observed in the retina of CTL eye (D).Scale bar: 20μm. E-F) Microphotograph of Hoechst (left, blue) and IBA1 (right, red) stained sections of a CPA treated eye (E) and Control eye (F). A lesser number of IBA1 positive cells are observed in the retina of CPA treated eye (E) while more cells are present in the retina of CTL eye. Scale bar: 20μm. G) Quantification of ONL TUNEL positive cells. CPA treatment produced a significant decrease of ONL positive nuclei when compared to CTL (1.454 ± 0.7376 vs 4.25 ± 1.379 apoptotic nuclei per 1000 μm2; unpaired Student´s t-test p<0.001; n = 8). ***p<0.001. H) Quantification of GFAP positive area staining. CPA treatment produced a significant decrease of GFAP expression when compared to CTL (13.02±10.67% vs control retinas 33.32±15.23%; unpaired Student´s t-test; p<0.01; n = 8). **p<0.01. I) Quantification of IBA1 positive cells. CPA treatment produced a significant decrease of IBA1 positive cells when compared to CTL (1.283 ± 0.1554 vs 2.683 ± 0.6115 IBA1 positive cells per 10000 μm2; unpaired Student´s t-test p<0.01; n = 4). **p<0.01.

No TUNEL positive nuclei were found in control eyes before illumination, but after 1 day of CI, apoptotic nuclei were found in retinal outer nuclear layer (ONL) in both experimental conditions (CPA and control). However, CPA treated retinas presented a lower number of TUNEL positive nuclei in the outer nuclear layer than control animals (Fig 2A and 2B). Quantification by image analysis showed an average of 1.454 ± 0.737 apoptotic nuclei per 1000 μm^2^ in the outer nuclear layer of CPA treated retinas vs 4.25 ± 1.379 apoptotic nuclei per 1000 μm^2^ in the outer nuclear layer of control retinas. The difference was significant using an unpaired Student´s t-test (p< 0.001; n = 8) (Fig 2G).

Before illumination, GFAP immunoreactivity was restricted to the end feet of Müller cells close to the inner limiting membrane. After illumination GFAP immunoreactivity increased in Müller cell processes across the whole retinas and a strong staining was observed in the end feet close to the inner limiting membrane in both conditions. However, in animals treated with CPA, Müller cell processes were thinner and GFAP immunoreactivity of the ending feet was weaker compared with control, indicating lower levels of glial activation (Fig 2C and 2D). In fact, image analysis quantification showed a significant decrease of GFAP positive area in CPA treated retinas (13.02 ± 10.67%) vs control retinas (33.32 ± 15.23%) (unpaired Student´s t-test; p<0.01; n = 8) (Fig 2H).

**Fig 3:**
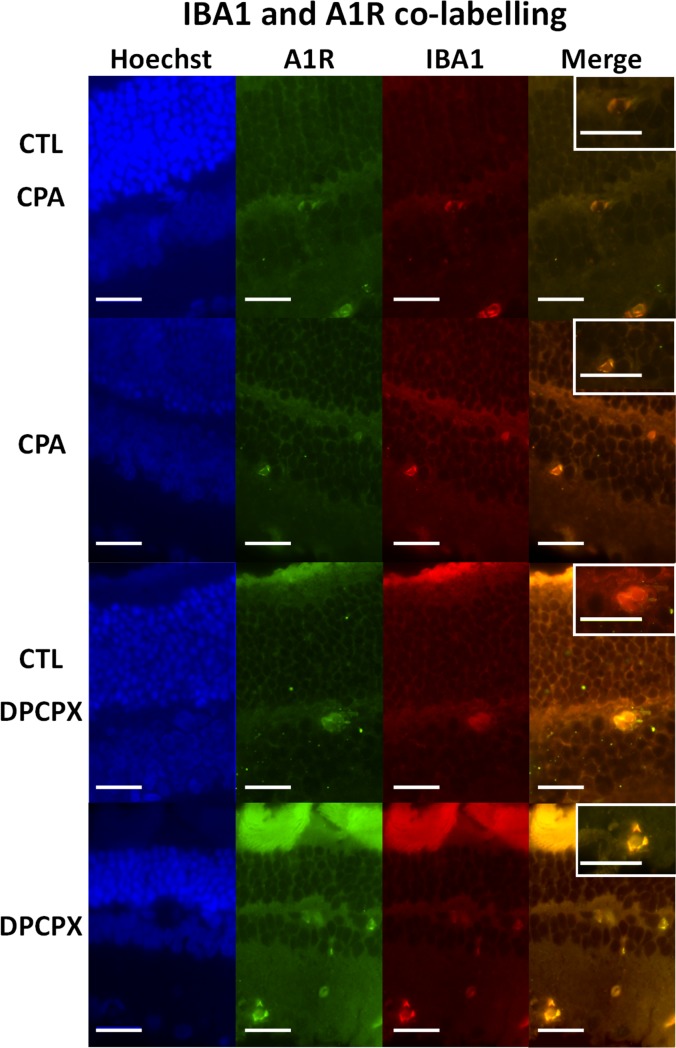
Double immunolabeling for Iba 1 and A1 receptor. Representative sections of CPA Control retina (top row); CPA treated retina (second row); DPCPX Control retina (third row) and DPCPX treated retina (fourth row). In every case nuclear staining with Hoechst 33258 (blue), A1 receptor immunolabeling (green); Iba 1 immunolabeling (red), and double labeling of the same sections may be observed form left to right. Insets show higher magnifications of merge images. Scale bars = 20 μm.

**Fig 4:**
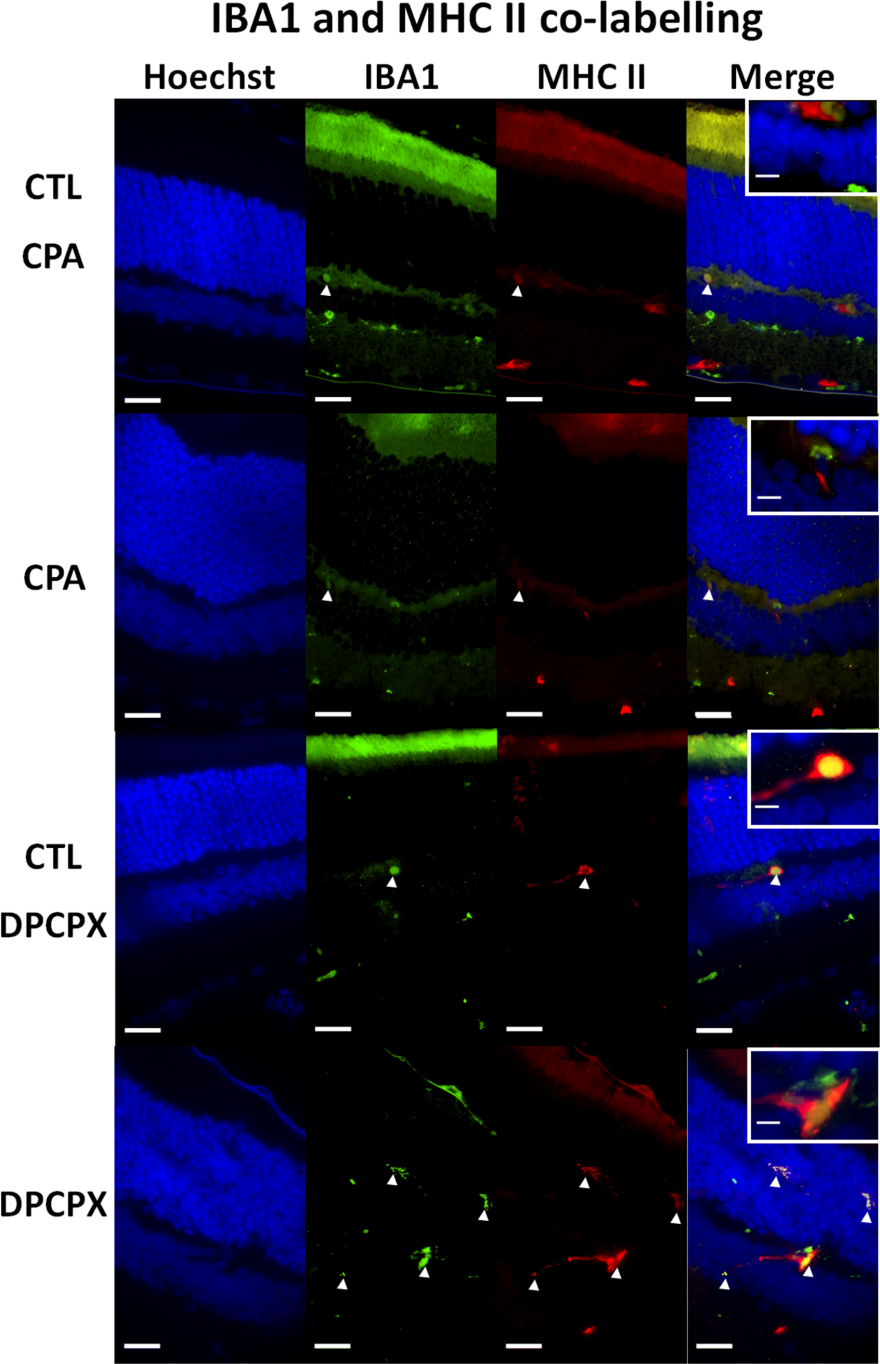
Double immunolabeling for Iba 1 and MHC-II. Representative sections of CPA Control retina (top row); CPA treated retina (second row); DPCPX Control retina (third row) and DPCPX treated retina (fourth row). In every case nuclear staining with Hoechst 33258 (blue), Iba 1 immunolabeling (green), MHC II immunostaining (red) and double labeling (merge) of the same sections may be observed form left to right. Insets show higher magnifications of merge images. Arrow heads show reactive microglial cells. Observe the low number of double stained reactive microglial cells in CPA treated retina and the higher number of double stained reactive microglial cells in DPCPX treated retina. Scale bars = 20 μm and 5μm (inset).

**Fig 5:**
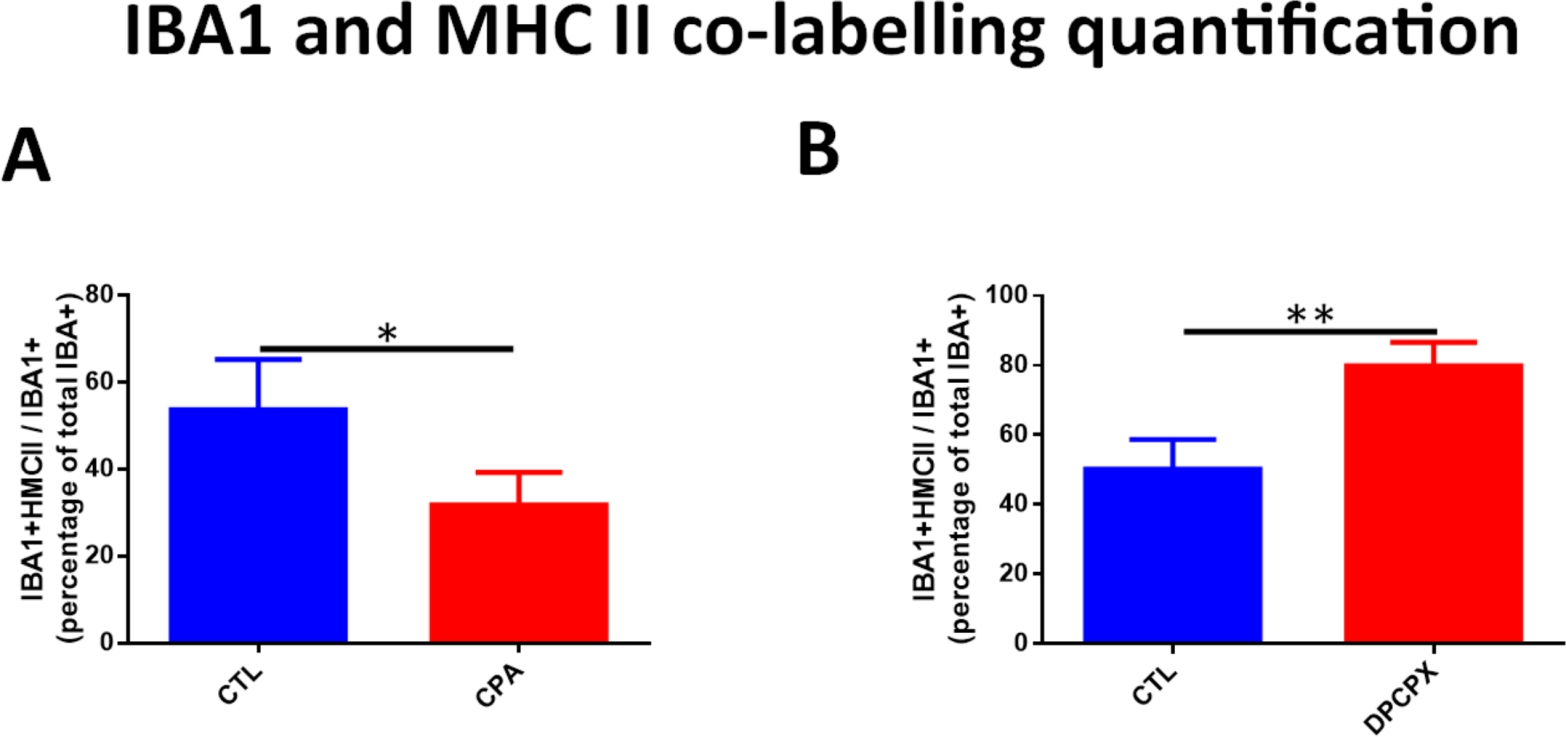
Quantification of double immunolabeling for Iba 1 and MHC-II. The number of activated microglia (Iba 1^+^/ MHC-II^+^) was expressed as the percentage of the total number of Iba 1+ microglial cells per section. CPA treated retinas showed a significant decrease of reactive microglial cells (p<0.05) while DPCPX treated retinas showed a highly significant increase of reactive microglial cells (p<0.01).

CPA treated retinas showed a significant decrease in the number of Iba 1 positive microglial cells (Fig 2E and 2F). Image analysis quantification showed that the decrease was significant (CPA: 1.28 ± 0.155 cells/10,000 µ^2^ vs CTL: 2.68 ± 0.61 cells/10,000 μ^2^, p<0.01) (Fig 2I). In both conditions, CPA and Control, double labeling technique using primary antibodies to A1 receptor and Iba 1 showed the co-localization of the A1 receptor and Iba1 on microglial cells (Fig 3, Top and second row, and Fig 4). In order to detect reactive microglia, double labeling technique using primary antibodies to Iba 1 and MHC-II was performed (Fig 4, Top and second rows). CPA treated retinas showed a significant decrease of the percentage of reactive microglial cells (Iba 1+ and MHC-II +) compared to control (p < 0.05) (Fig 5).

### DPCPX increases apoptotic cell death, glial reactivity and Iba 1^+^ /MHC-II^+^ microglial cells

**Fig 6:**
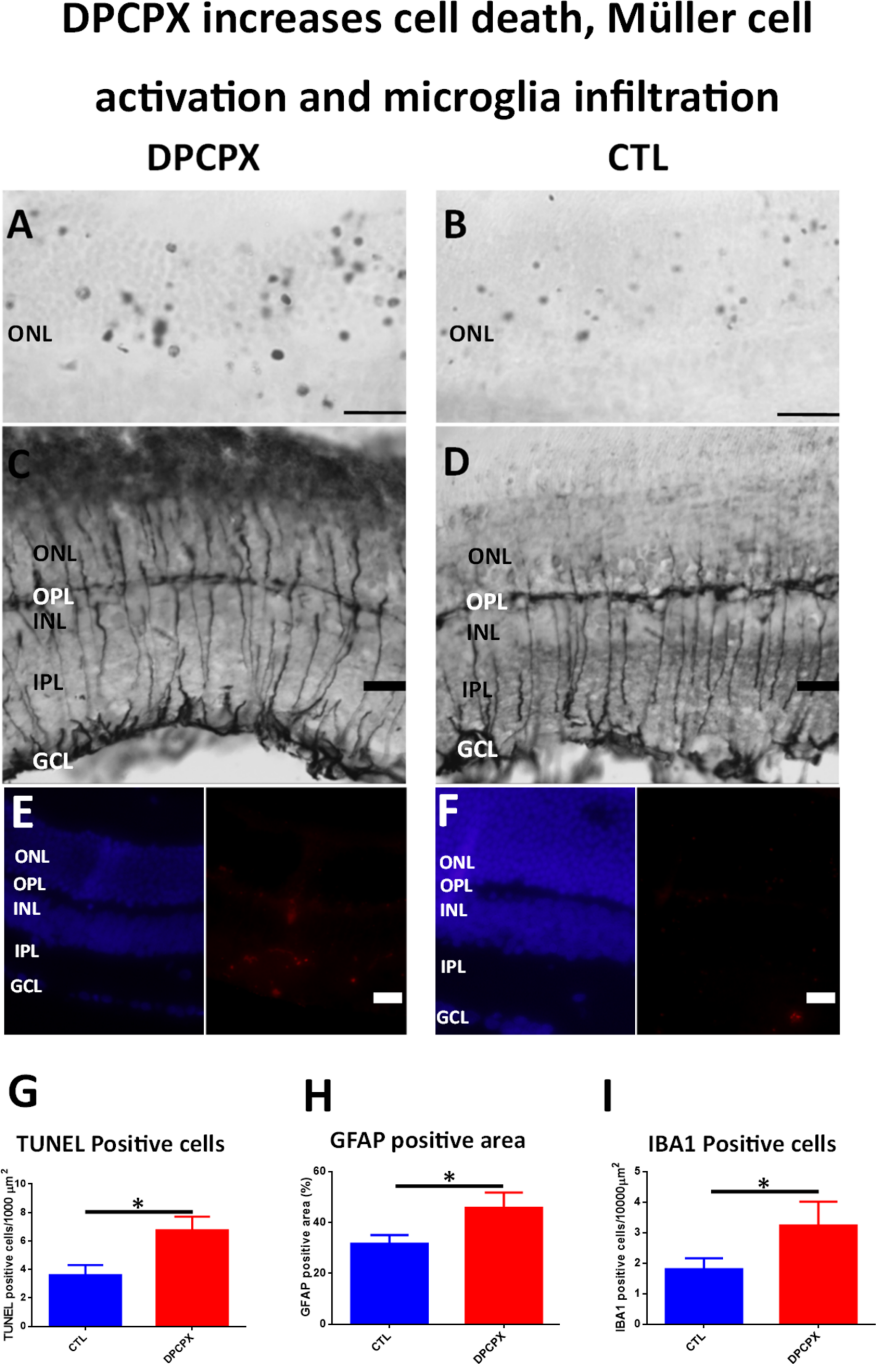
DPCPX increases cell death, Müller cell activation and microglial infiltration. A-B) Microphotograph of representative sections showing TUNEL staining of the outer nuclear layer of the retina of a DPCPX treated eye (A) and of a CTL eye (B). Observe the important number of apoptotic nuclei in DPCPX treated eye (A) compared to the number of apoptotic nuclei present in CTL eye (B). Scale bar: 20μm. C-D) Microphotograph of GFAP immunostained sections of the retina of a DPCPX treated eye (C) and of a control eye (D). Higher immunoreactivity of Müller cells are observed in the retina of DPCPX treated eye (C) compared to Müller cells in the retina of CTL eye (D).Scale bar: 20μm. E-F) Microphotograph of Hoechst (left, blue) and IBA1 (right, red) stained sections of a DPCPX treated eye (E) and Control eye (F). A higher number of IBA1 positive cells are observed in the retina of DPCPX treated eye (E) compared to those observed in the retina of CTL eye Scale bar: 20μm. G) Quantification of ONL TUNEL positive cells. DPCPX produced a significant rise in ONL positive nuclei when compared to CTL (6.755±2.337 vs 3.608±1.402; unpaired Student´s t-test; p<0.05; n = 8). *p< 0.05. H) Quantification of GFAP positive area staining. DPCPX produced a significant rise in GFAP expression when compared to CTL (45.75±16.1% vs 31.69±10.15%; unpaired Student´s t-test; P<0.05; = 4). *p< 0.05. I) Quantification of IBA1 positive cells. DPCPX treatment produced a significant increase of IBA1 positive cells when compared to CTL (3.235 ± 1.356 vs 1.801 ± 0.8941 IBA1 positive cells per 10000 μm2; unpaired Student´s t-test p<0.05; n = 4). *p<0.05.

In contrast with the results observed with CPA, after the illumination procedure a higher number of TUNEL positive nuclei was observed in the outer nuclear layer of DPCPX treated eyes versus control (Fig 6A and 6B). Quantification by image analysis showed an average of 6.755 ± 2.337 apoptotic nuclei per 1000 μm^2^ in the outer nuclear layer of DPCPX treated retinas vs 3.608 ± 1.402 apoptotic nuclei per 1000 μm^2^ in control retinas. The difference was significant using an unpaired Student´s t-test (p < 0.05; n = 8) (Fig 6G).

An increase in GFAP immunoreactivity was observed in DPCPX treated retinas compared to their controls (Fig 6C and 6D). In animals treated with DPCPX, Müller cell processes were thicker and their ending feet close to the inner limiting membrane were bigger and more intensely stained than those observed in control, indicating a rise of glial activation (compare Fig 6C and 6D). In fact, image analysis quantification showed a significant increase of the percentage of GFAP positive area in DPCPX treated retinas (45.75 ± 16.1%) vs their respective controls (31.69 ± 10.15%) (unpaired Student´s t-test; p = 0.05; n = 8) (Fig 6H).

DPCPX treated retinas showed a significant increase in the number of Iba 1 positive microglial cells compared to controls (Fig 6E and 6F). Image analysis quantification showed that the increase was significant (DPCPX: 3.235 ± 1.356 cells/10,000 μ^2^ vs CTL: 1.80 ± 0.89 cells/10,000 μ^2^, p< 0.05) (Fig 6I). In both conditions, DPCPX and Control (Fig 3, third and fourth row), double labeling technique using primary antibodies to A1 receptor and Iba 1 showed the co-localization of the A1 receptor and Iba1 on microglial cells. In order to assess reactive microglia, double labeling technique using primary antibodies to Iba 1 and MHC-II was performed (Fig 4, third and fourth rows). DPCPX treated retinas showed a highly significant increase of the percentage of reactive microglial cells (Iba 1^+^ and MHC-II ^+^) compared to control (p < 0.01) (Fig 5).

### Effect of CPA and DPCPX on activated Caspase 3 and GFAP expression by Western blot assays

**Fig 7:**
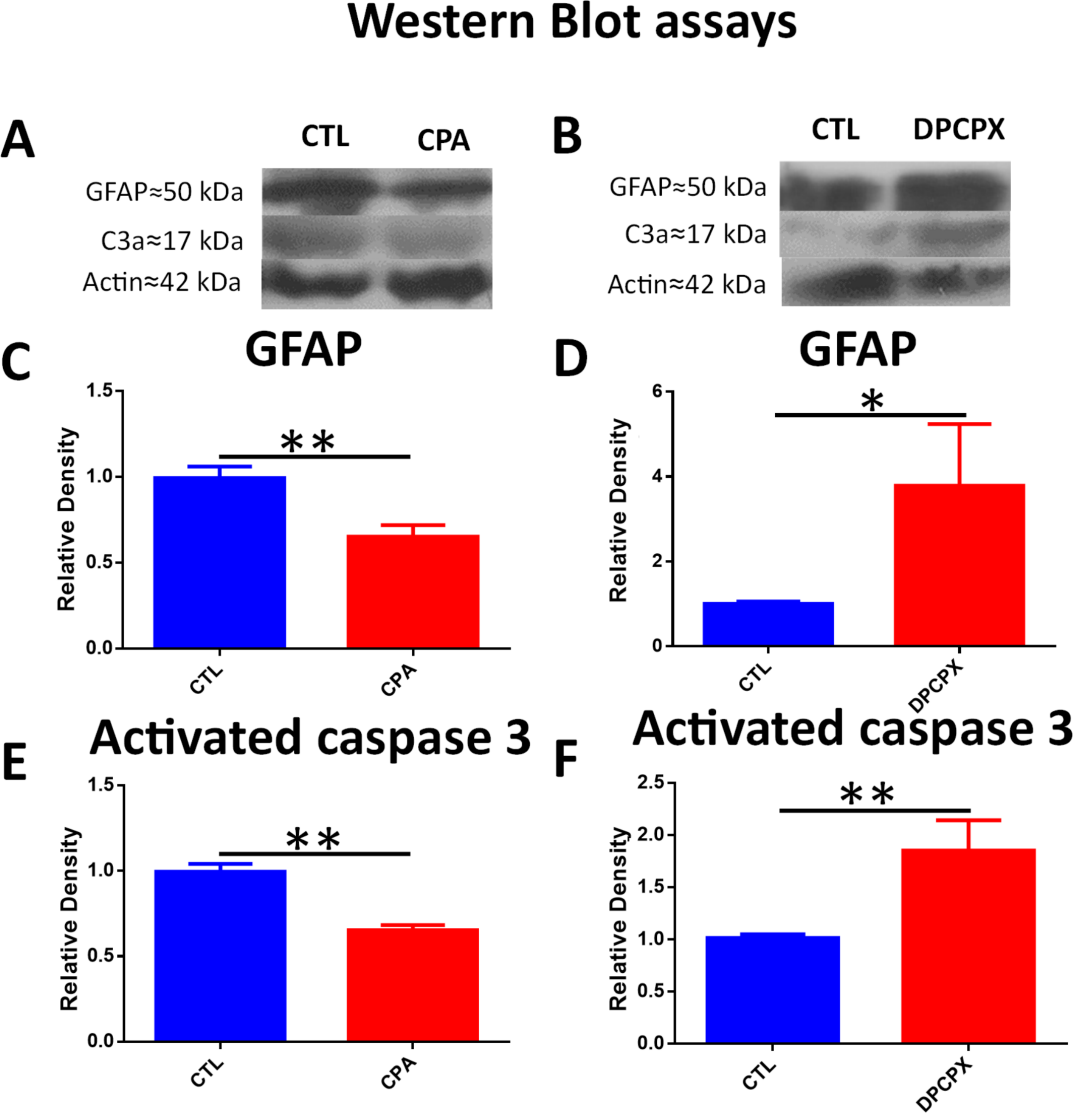
CPA treatment lowered activated caspase 3 and GFAP levels while DPCPX increased activated Caspase 3 and GFAP levels. A) Representative Western Blot of CPA and CTL treated eyes (cropped blots are displayed). From top to bottom bands correspond to GFAP, Actin and C3a. B) Representative Western Blot of DPCPX and CTL treated eyes (cropped gels/blots are displayed). From top to bottom bands correspond to GFAP, Actin and C3a. C) Quantification of GFAP by WB. CPA produced a highly significant decrease of GFAP relative density compared to CTL (0.652 ± 0.117 vs 0.993 ± 0.1329; unpaired t-test; p<0.01; n = 4), **p<0.01. D)Quantification of GFAP by WB. DPCPX produced a significant rise in GFAP relative density compared to CTL (3.785 ± 2.515 vs 1.00 ± 0.108; unpaired Student´s t-test; p<0.05; n = 4), *p< 0.05. E) Quantification of C3a by WB. CPA produced a highly significant decrease of C3a relative density compared to CTL (0.6527±0.03 vs 0.996±0.04; unpaired Student´s t-test; P = 0.001; n = 4), **p<0.01. F) Quantification of C3a by WB. DPCPX produced a highly significant rise in C3a relative density compared to CTL (1.85±0.5 vs 1.01±0.07; unpaired Student´s t-test; p<0.01; n = 4), **p<0.01.

In CPA treated eyes, lower levels of activated Caspase 3 protein were detected compared to control (CPA = 0.6527 ± 0.03 vs control = 0.996± 0.04; unpaired Student´s t-test; p< 0.01; t = 5.834; n = 4) (Fig 7A and 7E and S2 and S3 Figs).

GFAP protein levels were significantly lower in the CPA treated eyes (0.652 ± 0.117) than in control eyes (0.993 ± 0.1329) (unpaired Student´s t-test; p<0.01; t = 3.53; n = 4), (Fig 7A and 7C and S2 and S3 Figs).

The results of Western Blot assays were in accordance to those observed with TUNEL technique and immunohistochemistry. In DPCPX treated eyes, higher levels of activated Caspase 3 (1.85 ± 0.5 vs 1.01 ± 0.07; unpaired Student´s t-test; p<0.01; t = 3.385; n = 4), and GFAP (3.785 ± 2.515 vs 1.00 ± 0.108; unpaired Student´s t-test; p<0.05; t = 2.2885; n = 4) were found, thus confirming the presence of more apoptosis and an increase of glial reactivity, respectively (Fig 7B, 7D and 7F and S2 and S3 Figs).

### Effect of CPA and DPCPX on scotopic electroretinograms and oscillatory potentials

**Fig 8:**
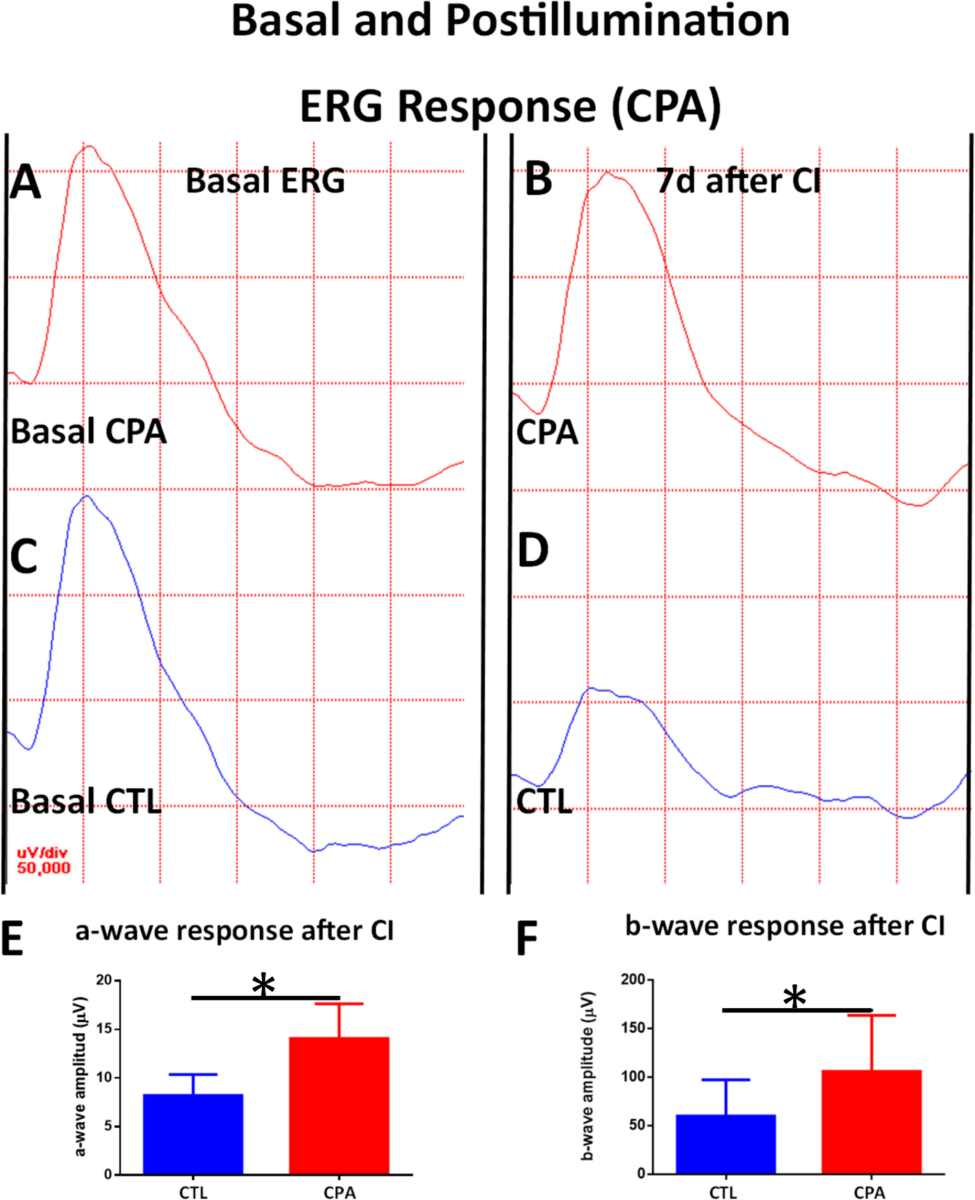
Effect of CPA treatment on ERG recordings (I): a-wave and b-wave. A) Basal ERG response of a CPA treated eye. B) ERG response a week after CI of a CPA treated eye. Observe a small increase of a-wave amplitude and the preservation of b-wave amplitude compared to Basal ERG (A). C) Basal ERG response of CTL eye. D) ERG response a week after CI of a CTL eye. Observe a decrease of both a-wave amplitude and b-wave amplitudes. E) Quantification of a-wave amplitude of both eyes a week after injection and 1d of CI. A significant higher amplitude of of a-wave was detected in CPA treated eyes compared to CTL eyes (14.07 ± 3.56 μV vs 7.14 ± O.63, unpaired Student´s t-test; p<0.05; n = 5), *p< 0.05. F) Quantification of b-wave amplitude of both eyes a week after injection and 1d of CI. A significant higher amplitude of b-wave was detected between CPA treated eyes compared to CTL eyes (106 ± 57.9 μV vs 60.11 ± 37.37 µV; unpaired Student´s t-test; p<0.05; n = 5), *p< 0.05.

**Fig 9:**
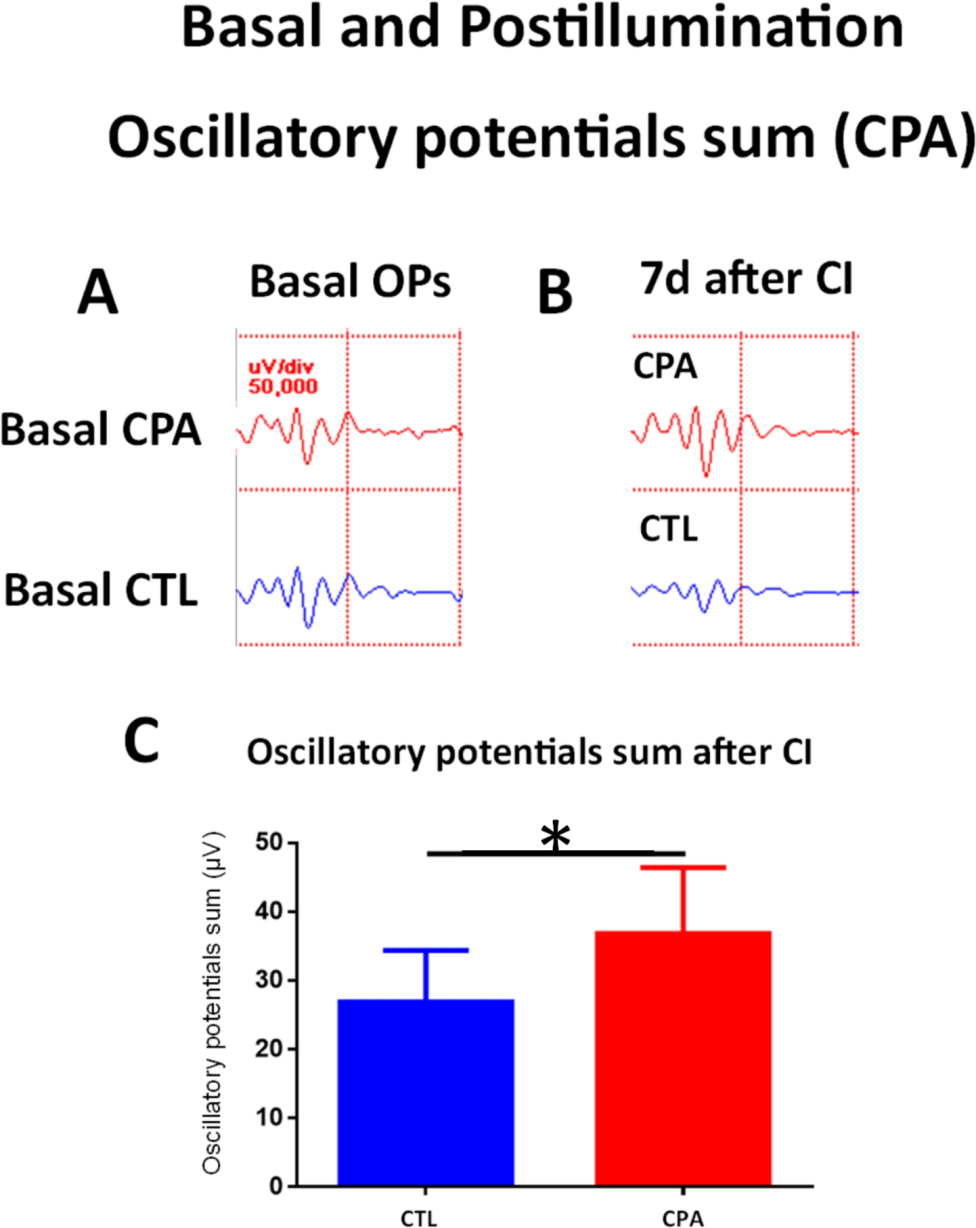
Effect of CPA treatment on ERG recordings (II): Oscillatory potentials. A) Basal Oscillatory potentials response of a CPA treated eye (top) and of a CTL eye (bottom). B) Oscillatory potentials response a week after after 1d of CI of a CPA treated eye (top) and of a CTL eye (bottom). C) Quantification of oscillatory potentials sum amplitude of both eyes (CTL and CPA) a week after injection and 1d of CI. A significant difference was detected between CPA and CTL eyes (36.87 ± 9.58 µV vs 26.88 ± 7.5 μV, unpaired Student´s t-test; p<0.05; n = 5), *p< 0.05.

A week after the CI exposure for 1 day, control eyes showed decreases in b-wave amplitude and oscillatory potential sum compared with their respective basal values (Fig 8C and 8D and Fig 9A and 9B). However, at the same time point, CPA treated eyes showed an increased amplitude for the a-wave and similar b-wave and oscillatory potentials compared to basal values measured before CI (Fig 8A and 8B and Fig 9A and 9B and Table 1).

**Table 1:** ERG recordings of control and CPA treated eyes.

	CONTROL EYE		CPA TREATED EYE	
	BASAL	ILLUMINATED	BASAL	ILLUMINATED
a-wave (μV)	10.61 ± 6.27	7.14 ±0.63	9.88 ± 3.38	14.07 ± 3.56*
b-wave (μV)	140.2 ± 72.16	60.11 ± 37.37	137.4 ± 50.53	106 ± 57.9*
OP (μV)	35.87 ± 13.56	26.88 ± 7.5	33.10 ± 7.18	36.87 ± 9.58*

Observe that recordings from CPA illuminated eyes differ significantly from control illuminated eyes (*, p<0.05).

When compared to control eyes, after exposure to continuous illumination, CPA treated eyes showed significantly higher amplitudes of all the electrophysiological parameters: a-wave (14.07 ± 3.56 µV vs 7.14 ± 0.63, unpaired Student´s t-test; p<0.05, t = 3.247) (Fig 8E), b-wave (106 ± 57.9 μV vs 60.11 ± 37.37 µV; unpaired Student´s t-test; p<0.05, t = 2.82) (Fig 8F), and oscillatory potential sum (Figs 7 and 9C) (36.87 ± 9.58 μV vs 26.88 ± 7.5 μV, unpaired Student´s t-test; p<0.05, t = 2.639).

In summary, continuous illumination induced an electrophysiological damage that was avoided by CPA treatment.

**Fig 10:**
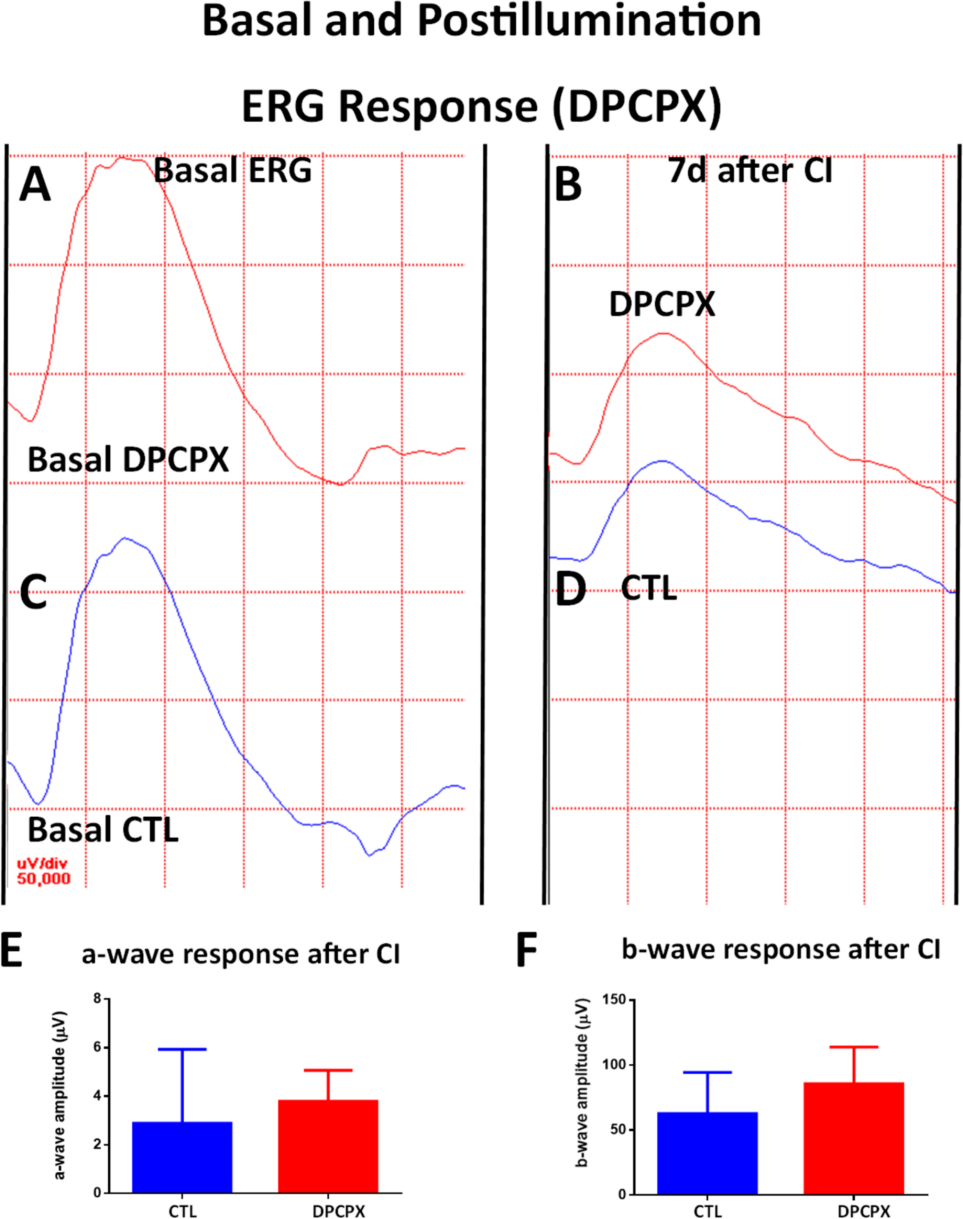
Effect of DPCPX treatment on ERG recordings (I): a-wave and b-wave. A) Basal ERG response of a DPCPX treated eye. B) ERG response a week after 1d of CI of a DPCPX treated eye. Observe a decrease in the amplitude of both a-wave and b-wave compared to Basal ERG (A). C) Basal ERG response of CTL eye. D) ERG response a week after CI of a CTL eye. Observe a decrease of both a-wave amplitude and b-wave amplitudes. E) Quantification of a-wave amplitude of both eyes a week after injection and 1d of CI. No significant difference was detected from basal levels after exposure to CI. F) Quantification of b-wave amplitude of both eyes a week after injection and 1d of CI. No significant difference was detected from basal levels after exposure to CI.

**Fig 11:**
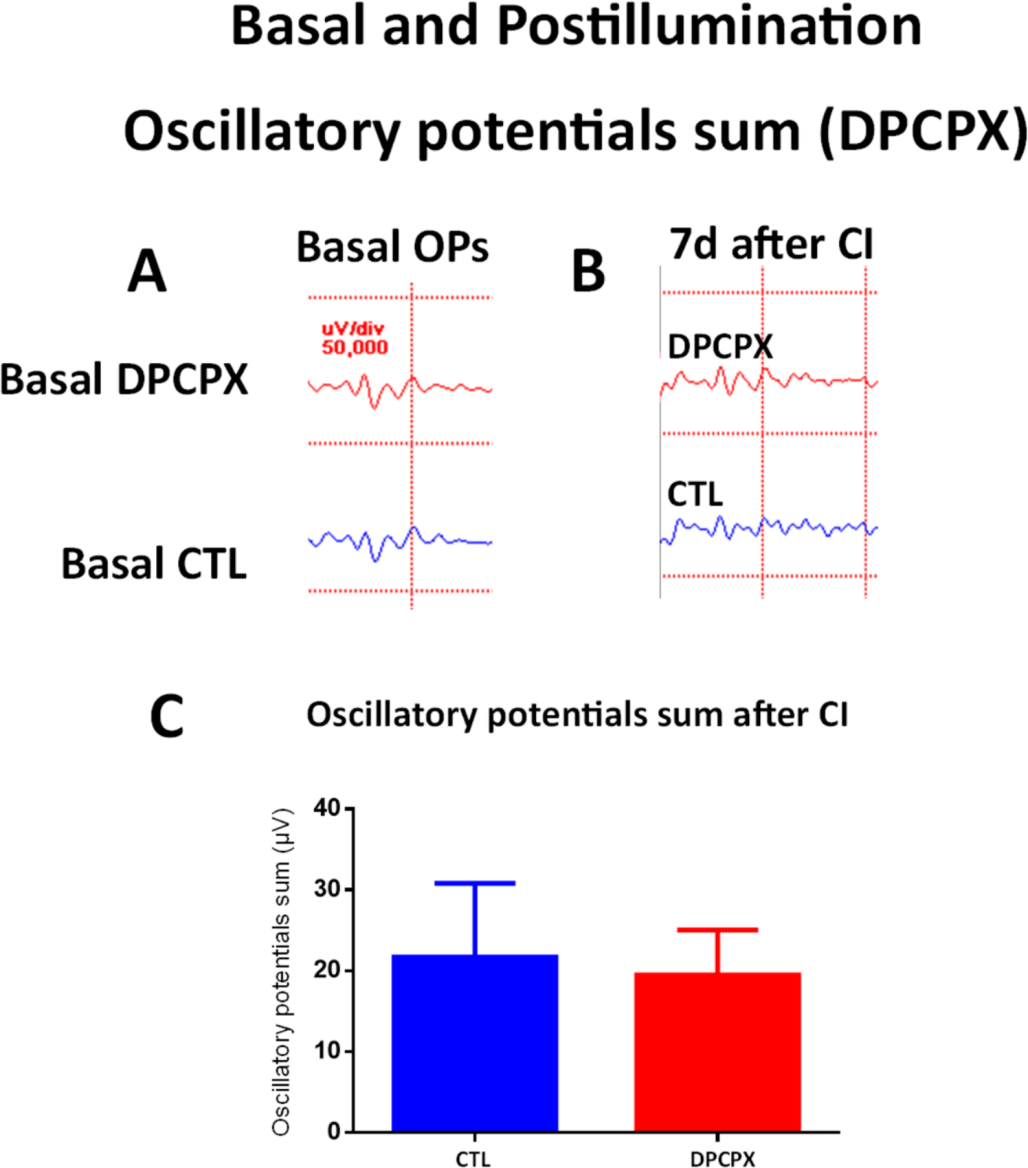
Effect of DPCPX treatment on ERG recordings (II): Oscillatory potentials. A) Basal Oscillatory potentials response of a DPCPX treated eye (top) and of a CTL eye (bottom). B) Oscillatory potentials response a week after 1d of CI of a DPCPX treated eye (top) and of a CTL eye (bottom).C) Quantification of oscillatory potentials sum amplitude of both eyes (DPCPX and CTL) a week after injection and 1d of CI. No significant difference was detected between DPCPX treated eyes and CTL eyes.

As mentioned above, a week after the continuous illumination exposure for 1 day, control eyes showed a decrease on the amplitude of the a-wave, b-wave (Fig 10C and 10D), and the oscillatory potentials sum (Fig 11A and 11B), compared with basal values measured before continuous illumination (Table 2).

**Table 2:** ERG recordings of control and DPCPX treated eyes.

	CONTROL EYE		DPCPX TREATED EYE	
	BASAL	ILLUMINATED	BASAL	ILLUMINATED
a-wave (μV)	10.61 ± 6.27	2.89 ± 3.04	9.38 ± 3.38	3.8 ± 1.27
b-wave (μV)	140.2 ± 72.16	62.81±31.66	137.4 ± 50.53	85.69 ± 28.21
OP (μV)	35.87 ±13.56	21.71 ± 9	33.10 ± 7.18	19.5 ± 5.54

Observe that recordings from DPCPX illuminated eyes did not differ significantly from control illuminated eyes.

After comparing DPCPX control eyes, illuminated for 1 day, with CPA control eyes illuminated for 1 day, a more important decrease of a-wave was observed which may be consequence of the drug vehicle (DMSO) which used to dissolve the DPCPX [45].

DPCPX treated eyes also showed decreases of the a-wave, b-wave, and oscillatory potential sum when compared to basal values measured before continuous illumination (Fig 10A and 10B, Fig 11A and 11B and Table 2).

When compared to control eyes after illumination, DPCPX eyes did not show significant differences in the amplitudes of the a-wave (Fig 10E, unpaired Student´s t-test, p = 0.61, t = 0.5486), b-wave (Fig 10F, unpaired Student´s t-test, p = 0.16, t = 1.079), or oscillatory potential sum (Fig 11C, unpaired Student´s t-test, p = 0.49, t = 0.02184).

In summary, illumination showed a deleterious effect on retinal function which was neither worsened nor prevented by DPCPX.

### Effect of CPA and DPCPX on the expression of nNOS, iNOS, IL-1β, TNFα and GFAP mRNAs

**Fig 12:**
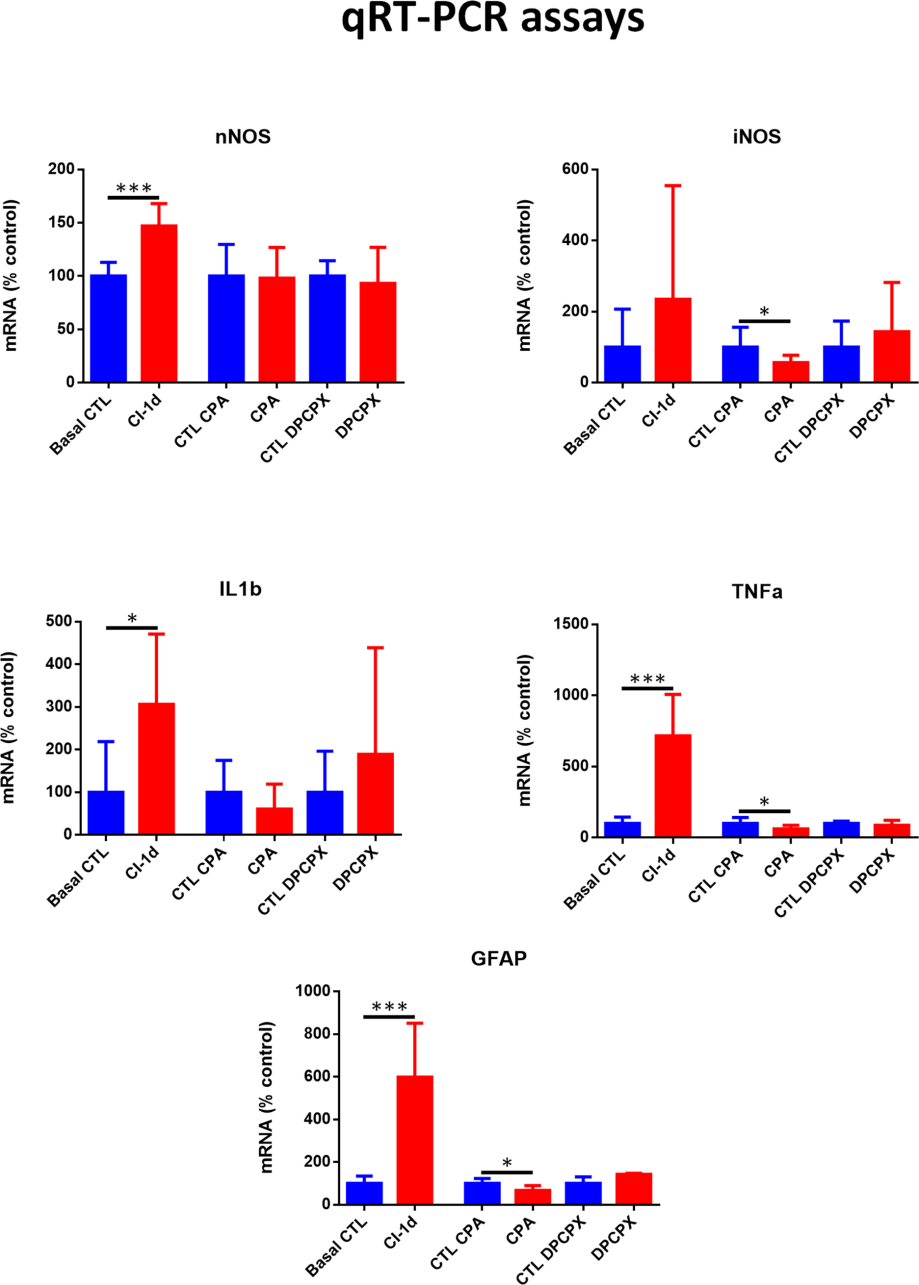
qRT-PCR of nNOS, iNOS, IL-1β and TNF α and GFAP mRNAs. Figures show mRNA expression of the retinas of unilluminated rats (Basal control), rats exposed to 1d of CI (CI 1d) and of rats treated with CPA or DPCPX or vehicle solutions (CTL CPA or CTL DPCPX) and then exposed to 1d of CI. Values are compared to their respective controls. Determinations were performed after exposure of the rat to one day of continuous illumination. A) nNOS, B) iNOS, C) IL-1β, D) TNF α and E) GFAP, bars represent mean ± SD, unpaired Student´s t-test, *p< 0.05, ***p<0.001.

Quantitave RT-PCR technique showed highly significant increases of nNOS, GFAP and TNFα mRNAs in non-treated rats exposed to 1d of CI compared to basal values (Fig 12). Also a significant increase of IL-1β mRNA was detected in this group but the method was unable to show a significant increase of iNOS. However, a significant decrease of iNOS mRNA expression was demonstrated in the retinas of CPA treated eyes compared to control (0.6990±0.4799 vs 1.322±0.7427, unpaired Student´s t-test, p<0.05, n = 5) while nNOS expression did not change (Fig 12). Also the levels of inflammatory cytokine TNFα significantly decreased in the retinas of CPA treated eyes compared to control (0.8903±0.4123 vs 1.510±0.6335; unpaired Student´s t-test, p<0.05, n = 5). GFAP mRNA expression was also diminished by CPA (0.7582±0.2721 vs 1.17±0.2728; unpaired Student´s t-test, p<0.05, n = 5). Levels of IL-1β did not change significantly (Fig 12). No significant changes were detected by qRT-PCR in any of the four genes studied when comparing the retinas of DPCPX treated eyes with their controls (Fig 12).

## Discussion

In the present work, we studied the effect of the intravitreal administration of an A1R agonist (CPA) and an A1R antagonist (DPCPX) on light induced retinal degeneration. Although a less invasive treatment could be implemented, intravitreal administration ensured achieving the intended drug concentration in the retinal tissue, as published [39, 40]. In patients suffering the wet variant of AMD, intravitreal injection is the common way of administrating the anti-VEGF treatment.

In our study, the decrease of TUNEL staining in the outer nuclear layer induced by CPA treatment clearly shows a neuroprotective role for A1 receptor agonists on photoreceptors. Neuroprotection is further confirmed by Western Blot analysis which shows a decrease of activated Caspase 3 levels. In addition, the results show a decrease of Müller cell activation as GFAP diminishes both by RT-PCR (mRNA), immunohistochemistry and Western Blot, supporting further evidence of a neuroprotective action through avoidance of glial reactivity. This effect may also be regarded as part of an antiinflammatory action. In fact, qRT-PCR results showed a significant diminution of the inflammatory cytokine TNFα and iNOS.

So, the administration of an A1 agonist shows a neuroprotective effect through mechanisms that prevented photoreceptor apoptotic cell death, a reduction of microglial response, demonstrated by a reduction in iNOS and TNFα mRNA expression, and a decrease of glial reactivity, as demonstrated by GFAP immunoreactivity, Western Blot and qRT-PCR. In order to confirm that CPA induced a reduction of microglial reactivity, retinas were stained with Iba 1 (ionized calcium adaptor molecule 1). Iba 1 is a microglial and macrophage-specific calcium-binding protein involved in the reorganization of actin cytoskeleton through Rac signaling pathway [46]. Iba 1 is involved in membrane ruffling and phagocytosis in activated microglia [47] and was previously used as a marker of reactive microglia after transient focal cerebral ischemia [48]. Our results showed a significant reduction of Iba1^+^ microglial cell population in CPA treated retinas while, on the opposite, DPCPX induced a highly significant increase of Iba 1^+^ microglial cells. Double labeling experiments showed the co-existence of A1R and Iba 1 demonstrating the direct effect of the agonists on microglial cells. As major histocompatibility complex class II (MHC-II) has been used to detect reactive microglia [36] we performed double labeling for Iba 1 and MHC-II. Our results showed a decrease of the reactive microglial cells (Iba 1^+^/MHC-II^+^) in CPA treated retinas compared to controls and an increase of reactive microglial cells (Iba 1^+^/MHC-II^+^) in DPCPX treated retinas compared to controls. These results are in agreement with previous reports that showed that the activation of A1 receptor inhibits the morphological activation of microglia [49] and attenuates neuroinflammation and demyelination in a model of multiple sclerosis [50].

In a similar way, the blockade of A2A receptor in an animal model of ischemia reperfusion attenuated microglial reactivity and the increased expression and release of proinflammmatory cytokines and afforded protection to the retina [36, 37].

Although microglia is involved in the inflammatory reaction in the retina producing inflammatory cytokines as TNFα, other sources of TNFα may be other resident activated macrophages, as well as CD4+ lymphocytes and natural killer cells which arrive to the retinal tissue by the blood vessels. Also Müller cells and retinal pigmented cells have been reported to produce TNFα in autoimmune uveoretinitis [51] so these cells may also contribute to the inflammatory response and their role cannot be ruled out.

The changes in ERG response support the idea that A1 modulation impacts not only on photoreceptor survival but also on the functionality of photoreceptors themselves and of other inner retina cell types (mainly bipolar and ganglion cells) as a-wave, b-wave, and oscillatory potentials were protected by CPA pretreatment. On the contrary, DPCPX, an A1R antagonist, worsened biochemical parameters and two of the studied morphological parameters (apoptotic nuclei and GFAP area). In addition, A1 antagonist, DPCPX, was unable to alter gene expression of iNOS, nNOS or inflammatory cytokines IL-1β and TNFα. It may be speculated that higher doses of DPCPX, or a longer time of exposure to the drug may alter retinal physiology. An alternative explanation may be that the A1R antagonist, DPCPX, lacks its effect in the absence of an increased A1 receptor activity which could play a part in the CI model pathophysiology.

The obtained results are in accordance with other reports on the role of adenosine in retinal neuroprotection mediated by A1 or A2A receptors [38, 35].

However, other questions remain to be answered, such as how the changes in A1R activation are connected with the apoptosis of photoreceptors, inflammation and glial reactivity.

In the model of LIRD, the administration of an A1R agonist could protect the retina through the presynaptic inhibition of glutamate release and the modulation of NMDA receptor activity as was previously demonstrated in rat hippocampus [52].

In rod photoreceptors, the observed neuroprotective effect of CPA could be mediated by the inhibition of calcium influx as it is known that adenosine inhibits calcium influx through L-type calcium channels [53]. Also the observed protective effect of CPA on photoreceptors could be mediated by its antioxidant effect as CPA inhibits lipid peroxidation and potentiates the antioxidant defense mechanisms (peroxidase and catalase enzymes) [54]. In addition, the activation of A1 receptors inhibits adenylate cyclase (AC) and decrease intracellular cAMP concentration. These changes decrease cell metabolism and neuronal energy requirements enhancing cell survival [54, 55].

Adenosine transmission also plays a role directly on the immune response. Higher A1 activity is necessary to diminish the immune response and promote cell survival [56]. So, we speculate that the neuroprotective role of CPA in LIRD could also be mediated through an effect on the immune response as well. Although the immune response is a late event in other models of retinal degeneration, our results clearly showed that CPA induced a significant decrease of Iba 1 reactive microglial cell population, and a decrease of iNOS and TNF α mRNAs in this model of light induced retinal degeneration. Besides, IL-1β is responsible of triggering glial reactivity [57, 58] which was decreased in our model of LIRD by the treatment with CPA.

In addition, adenosine transmission works in coordination with other signalling systems that involve the production of trophic factors. A complex crosstalk between IL-6, A1R, and A2AR stimulates BDNF production and has been shown to protect retinal ganglion cells in vitro [59, 60].

A cardiovascular effect could also be involved among the neuroprotective mechanisms mediated by adenosine A1 receptors, as was demonstrated in retinal ischemic insults that adenosine induces hyperhemia that protects neurons from glutamate toxicity [34].

As consequence of our findings a new strategy using A1 agonists could be used to prevent retinal degeneration. Knowing that AMD disease starts in one eye and usually progresses to the other one; the second eye could be protected after the diagnosis. However, Adenosine receptors can be found in most cells, widely distributed through the body, so the agonist will act not only on cells involved in the disease but also on cells involved in different physiological processes [61].

As adenosine receptors are present in most cells, and agonists have adverse effects, including sedation, headache, vasodilation, atrioventricular block, and bronchoconstriction [62, 63]; therapeutic strategies should target these receptors only when and where agonists are needed [61]. In order to do this we considered that CPA locally administered (intravitreal injection) is the best option, producing less collateral effects. The same concept is behind actual treatments of AMD that also use intravitreal injections of monoclonal antibodies against VEGF.

Although, in our study the administration of CPA was given preventively before illumination, it could be administered after illumination to treat retinal degeneration but further studies are needed to confirm if it is useful as a therapeutic agent in this case.

The present study shows evidence supporting that adenosine, acting through A1 receptors, is an important factor in degenerative diseases of the eye and its modulation may be used as a neuroprotective strategy. However, a single treatment with CPA, an A1 agonist, reported here did not accomplish a total prevention of retinal degeneration. Thence a repetition of the treatment could be considered as well as a combination with other drugs and/or trophic factors. Although further work is needed to confirm our hypothesis, the modulation of A1 receptor has a translational value as it could be a useful strategy to prevent the progression of AMD and other degenerative diseases in humans.

In this work we have shown that a single pharmacological intervention previous to the beginning of the photic damage was able to swing the retinal fate in opposite directions. While CPA, an A1 agonist, shows a retinal neuroprotective effect; DPCPX, an A1 antagonist, worsened many of the parameters chosen to assess damage. These results propose a protective role for A1 activation in LIRD in accordance with other models of retinal degenerative diseases.

Furthermore, LIRD is a valid model for an acquired degenerative disease of the outer retina, since it recapitulates many of the human symptoms of AMD, such as non-classic transmission and its pleiotropic effects on different cell types involved in inflammation, apoptotic cell death and normal neuronal function.

In summary, adenosine and the activation of the A1 receptor are promising targets to accomplish neuroprotection in LIRD and, hopefully, in retinal degenerative diseases.

## Supporting information

S1 Fig Negative controls of double immunolabeling for Iba 1/A1R (top row) and for Iba 1/MHC-II (second row). Representative sections of Control retinas in which primary antibodies were omitted. In every case sections were incubated with, goat anti rabbit antibody conjugated to Alexa Fluor® 488 and goat anti-mouse antibody conjugated to Alexa Fluor® 555. In every case background images are shown as well as their corresponding merge images. Scale bars = 20 μm.
(TIF)

S2 Fig Representative Western blots of CPA, DPCPX treated eyes and their respective controls. (TIF)

S3 Fig Representative Western blots of CPA, DPCPX treated eyes and their respective controls. (TIF)
