# Supplementary figures and images for: Adenosine A1 receptor: A neuroprotective target in light induced retinal degeneration
Source: PLoS One. 2018 Jun 18;13(6):e0198838. doi: 10.1371/journal.pone.0198838 (PMC6005487; doi:10.1371/journal.pone.0198838)

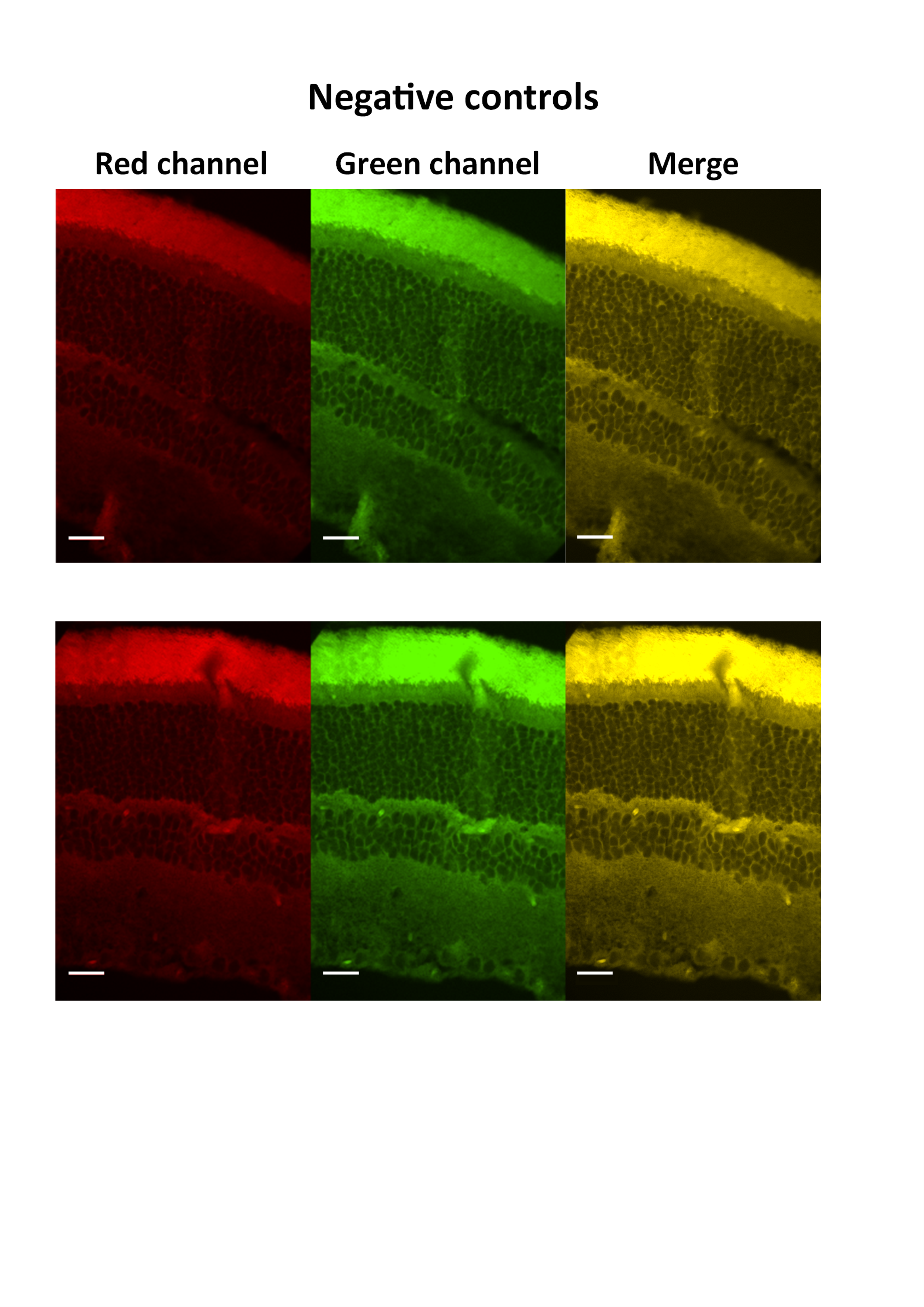

Supplement: S1 Fig — Representative sections of Control retinas in which primary antibodies were omitted. In every case sections were incubated with, goat anti rabbit antibody conjugated to Alexa Fluor® 488 and goat anti-mouse antibody conjugated to Alexa Fluor® 555. In every case background images are shown as well as their corresponding merge images. Scale bars = 20 μm. (TIF) [file pone.0198838.s001.tif]

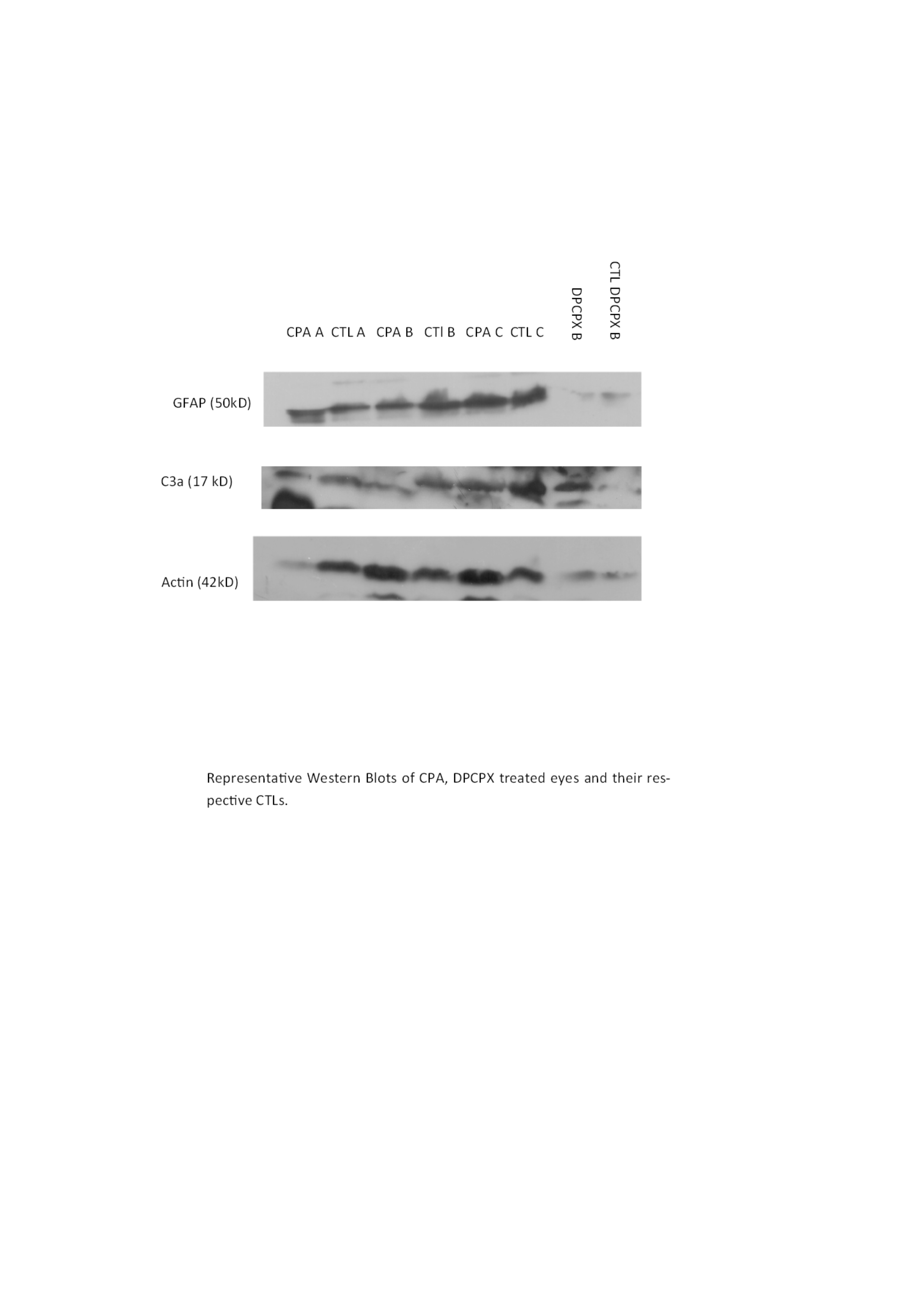

Supplement: S2 Fig — (TIF) [file pone.0198838.s002.tif]

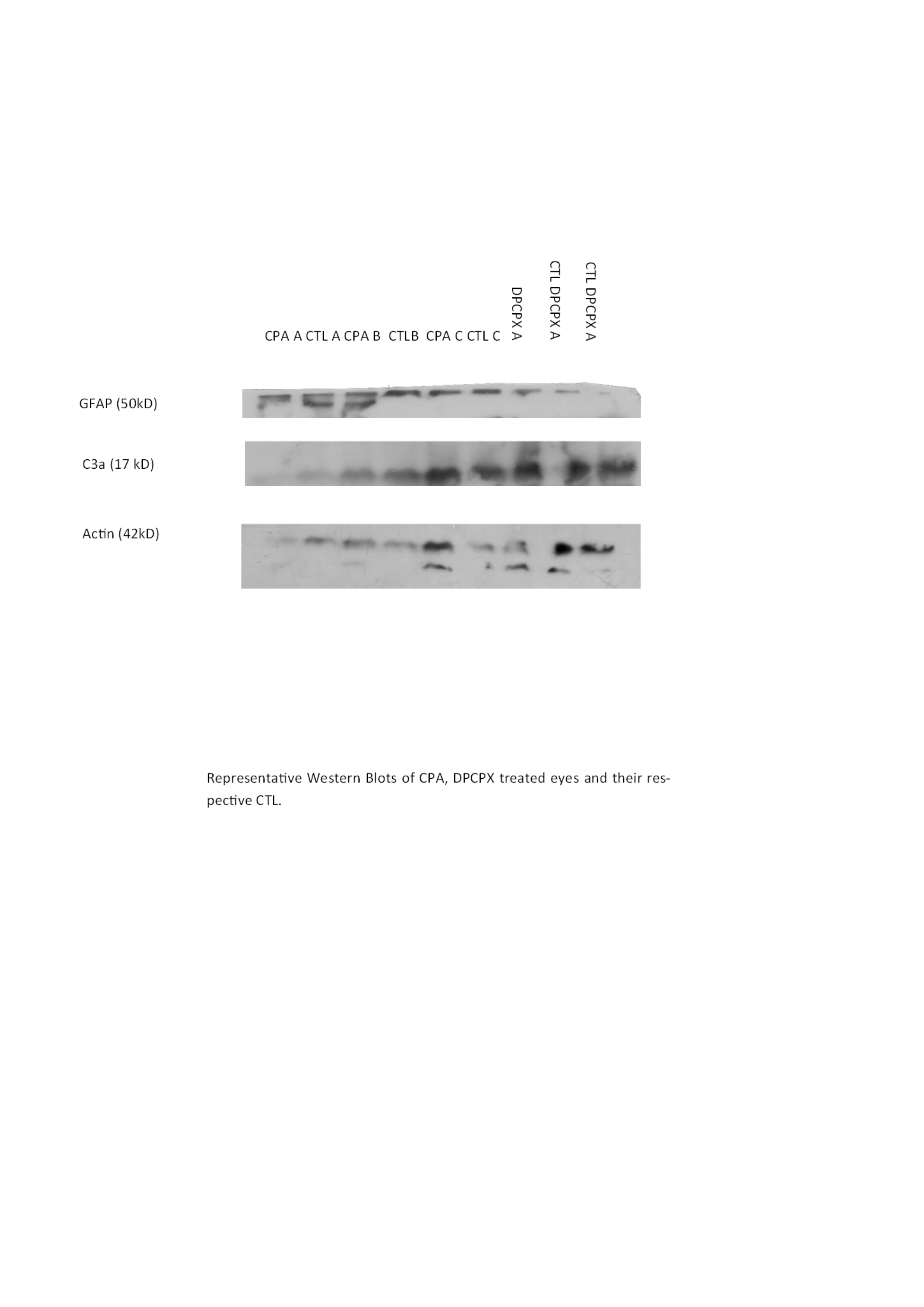

Supplement: S3 Fig — (TIF) [file pone.0198838.s003.tif]
